# A key to the North American genera of Stipeae (Poaceae, Pooideae) with descriptions and taxonomic names for species of *Eriocoma*, *Neotrinia*, *Oloptum*, and five new genera: *Barkworthia*, ×*Eriosella*, *Pseudoeriocoma*, *Ptilagrostiella*, and *Thorneochloa*

**DOI:** 10.3897/phytokeys.126.34096

**Published:** 2019-07-16

**Authors:** Paul M. Peterson, Konstantin Romaschenko, Robert J. Soreng, Jesus Valdés Reyna

**Affiliations:** 1 Department of Botany MRC-166, National Museum of Natural History, Smithsonian Institution, Washington, DC 20013-7012, USA National Museum of Natural History, Smithsonian Institution Washington United States of America; 2 Departamento de Botánica, Universidad Autónoma Agraria Antonio Narro, Saltillo, C.P. 25315, México Universidad Autónoma Agraria Antonio Narro Saltillo Mexico

**Keywords:** *Barkworthia*, *Eriocoma*, ×*Eriosella*, Gramineae, *Neotrinia*, North America, Poaceae, *Pseudoeriocoma*, *Ptilagrostiella*, Stipeae, taxonomy, *Thorneochloa*

## Abstract

Based on earlier molecular DNA studies we recognize 14 native Stipeae genera and one intergeneric hybrid in North America. We provide descriptions, new combinations, and 10 illustrations for species of *Barkworthia***gen. nov.**, *Eriocoma*, *Neotrinia*, *Oloptum*, *Pseudoeriocoma***gen. nov.**, *Ptilagrostiella***gen. nov.**, *Thorneochloa***gen. nov.**, and ×*Eriosella***nothogen. nov.** The following 40 new combinations are made: *Barkworthiastillmanii*, *Eriocomaalta*, *E.arida*, *E.arnowiae*, *E.bloomeri*, *E.bracteata*, *E.contracta*, *E.coronata*, *E.curvifolia*, *E.hendersonii*, *E.latiglumis*, *E.lemmonii*, E.lemmoniissp.pubescens, *E.lettermanii*, *E.lobata*, *E.nelsonii*, E.nelsoniissp.dorei, *E.nevadensis*, *E.occidentalis*, E.occidentalisssp.californica, E.occidentalisssp.pubescens, *E.parishii*, E.parishiissp.depaupertata, *E.perplexa*, *E.pinetorum*, *E.richardsonii*, *E.robusta*, *E.scribneri*, *E.swallenii*, *E.thurberiana*, *E.wallowaensis*, ×*Eriosellacaduca*, *Pseudoeriocomaacuta*, *P.constricta*, *P.editorum*, *P.eminens*, *P.hirticulmis*, *P.multinodis*, *Ptilagrostiellakingii*, and *Thorneochloadiegoensis*. A key to the native and introduced genera of North American Stipeae, and an overview of the tribe in North America and worldwide are given. Lectotypes are designated for *Eriocomacuspidata* Nutt., *Fendleriarhynchelytroides* Steud., *Stipabloomeri* Bol., *Stipacoronata* Thurb., *Stipamembranacea* Pursh, *Stipamormonum* Mez, *Stiparichardsonii* Link, and *Stipawilliamsii* Scribn. *Achnatherum* s.s. and *Piptatherum* s.s. are now restricted to Eurasia and the Mediterranean/Asia, respectively.

## Abstract (es)

Basados en estudios anteriores de ADN molecular, reconocemos 14 géneros nativos de Stipeae y un híbrido intergenérico en América del Norte. Se presentan descripciones, nuevas combinaciones, y 10 ilustraciones para las especies de *Barkworthia***gen. nov.**, *Eriocoma*, *Neotrinia*, *Oloptum*, *Pseudoeriocoma***gen. nov.**, *Ptilagrostiella***gen. nov.**, *Thorneochloa***gen. nov.** y ×*Eriosella***nothogen. nov.** Se realizan las siguientes 40 nuevas combinaciones: *Barkworthiastillmanii*, *Eriocomaalta*, *E.arida*, *E.arnowiae*, *E.bloomeri*, *E.bracteata*, *E.contracta*, *E.coronata*, *E.curvifolia*, *E.hendersonii*, *E.latiglumis*, *E.lemmonii*, E.lemmoniissp.pubescens, *E.lettermanii*, *E.lobata*, *E.nelsonii*, E.nelsoniissp.dorei, *E.nevadensis*, *E.occidentalis*, E.occidentalisssp.californica, E.occidentalisssp.pubescens, *E.parishii*, E.parishiissp.depaupertata, *E.perplexa*, *E.pinetorum*, *E.richardsonii*, *E.robusta*, *E.scribneri*, *E.swallenii*, *E.thurberiana*, *E.wallowaensis*, ×*Eriosellacaduca*, *Pseudoeriocomaacuta*, *P.constricta*, *P.editorum*, *P.eminens*, *P.hirticulmis*, *P.multinodis*, *Ptilagrostiellakingii* y *Thorneochloadiegoensis*. Se presenta una clave para los géneros nativos e introducidos de las especies norteamericanas, y una visión general de la tribu en América del Norte y en todo el mundo. Se designan lectotipos para *Eriocomacuspidata* Nutt., *Fendleriarhynchelytroides* Steud., *Stipabloomeri* Bol., *Stipacoronata* Thurb., *Stipamembranacea* Pursh, *Stipamormonum* Mez, *Stiparichardsonii* Link y *Stipawilliamsii* Scribn. *Achnatherum* s.s. y *Piptatherum* s.s. ahora están con distribución restringida- a Eurasia y el Mediterráneo/Asia, respectivamente.

## Introduction

The tribe Stipeae Dumort. comprises temperate, cool-season (C_3_) grasses that generally occupy somewhat moist to predominantly dry open grasslands and steppe communities in all continents except Antarctica. They represent an ecologically and morphologically specialized lineage within the subfamily Pooideae including approximately 527 species in 28 genera (Tzvelev 1977; Watson and Dallwitz 1992, Barkworth 2007; Romaschenko et al. 2008, 2010, 2011, 2012, 2013; Soreng et al. 2015, 2017). Historically, delimitation of taxa within the American Stipeae was based on broad concepts of the genera *Stipa* L. and *Oryzopsis* Michx. Hitchcock (1935, 1951) accepted three native genera in North America: *Oryzopsis* (12 spp.), *Piptochaetium* J. Presl (1 sp.), and *Stipa* (34 spp. + 2 introduced), and *Nassella* (Trin.) E. Desv. (1 sp. as introduced). In the Flora of North America Barkworth (2007) recognized nine native Stipeae genera: *Achnatherum* P. Beauv., *Amelichloa* Arriaga & Barkworth, *Hesperostipa* (M.K. Elias) Barkworth, *Jarava* Ruiz & Pav., *Nassella*, *Oryzopsis*, *Piptatherum* P. Beauv., *Piptochaetium* J. Presl, *Ptilagrostis* Griseb., and a single hybrid genus ×*Achnella* Barkworth. Recent molecular DNA studies have greatly increased our understanding of the evolutionary relationships among members of this tribe worldwide and in North America. In addition to the genera listed previously for North America, we now recognize species belonging to *Pappostipa* (Speg.) Romasch., P.M. Peterson & Soreng, *Patis* Ohwi, and *Piptatheropsis* Romasch., P.M. Peterson & Soreng (Romaschenko et al. 2008, 2011, 2012). Molecular phylogenetic study of the Stipeae using nine plastid and nuclear ITS DNA markers identified well-supported clades for ‘Stillmania’ (= *Barkworthia* gen. nov.), *Eriocoma* Nutt., ‘Miliacea’ (= *Oloptum* Röser & H.R. Hamasha), ‘Neotrinia’ [= *Neotrinia* (Tzvelev) M. Nobis, P. Gudkova & A. Nowak; Nobis et al. 2019], *Pseudoeriocoma* gen. nov., *Ptilagrostiskingii* (Bol.) Barkworth (= *Ptilagrostiella* gen. nov.) sister to the *Piptatheropsis* clade, and *Thorneochloa* gen. nov. (Hamasha et al. 2012; Romaschenko et al. 2012, 2013, 2014; Valdés Reyna et al. 2013). Table 1 provides an overview of the numbers of species in each Stipeae genus as applied in North America by Hitchcock (1951), Barkworth (2007), here, and Worldwide (updating Soreng et al. 2017).

**Table 1.** Overview of numbers of species in each genus of Stipeae in North America north of Mexico (FNA), endemic to Mexico, and Worldwide with distribution.

Genus	Year Publ.	Hitchcock 1951	Barkworth 2007	Present in FNA Region	Mexico Endemic	World-wide	Distribution (* = genus introduced in NA, ^c^ = cultivated NA)
*Achnatherum*	1812	0	28	0	0	21	Mediterranean & Eurasia
*Aciachne*	1881	0	0	0	0	3	South America
*Amelichloa*	2006	‒	3	3	0	5	Americas
*Anatherostipa*	1996	‒	0	0	0	8	South America
*Anemanthele*	1985	‒	0	1	0	1	New Zealand*^c^
*Austrostipa*	1996	‒	2	2	0	64	Australia*^c^
*Barkworthia*	*here*	‒	‒	1	0	1	United States
*Celtica*	2004	‒	1	1	0	1	Mediterranean*^c^
*Eriocoma*	1818	0	‒	25	2	27	North America
*Hesperostipa*	1993	‒	4	4	1	5	North America
*Jarava*	1794	0	3	1	1	33?	Latin America*^c^
*Lorenzochloa*	1969	‒	0	0	0	1	South America
*Macrochloa*	1829	0	1	1	0	1	Mediterranean*^c^
*Nassella*	1854	1	10	10	3	117	Americas
*Neotrinia*	2019	‒	‒	1	0	1	Asia *^c^
*Oloptum*	2012	‒	‒	1	0	1	Mediterranean*^c^
*Ortachne*	1854	0	0	0	0	2	South America
*Orthoraphium*	1841	0	0	0	0	1	Southeast Asia
*Oryzopsis*	1803	12	1	1	0	1	North America
*Pappostipa*	2008	‒	‒	2	0	31	South America*^c^
*Patis*	1942	0	0	1	0	3	East Asia & North America
*Piptatheropsis*	2011	‒	‒	5	0	5	North America
*Piptochaetium*	1830	1	6	6	5	35?	Americas
*Piptatherum*	1812	0	7	0	0	32	Mediterranean and Asia
*Psammochloa*	1927	0	0	0	0	1	East Asia
*Pseudoeriocoma*	*here*	‒	‒	1	5	6	North America
*Ptilagrostiella*	*here*	‒	‒	1	0	1	United States
*Ptilagrostis*	1852	0	2	1	0	15	East Asia & North America
*Stipa*	1753	34 + 2	2	1	0	150+	Mediterranean & Eurasia*^c^
*Stipellula*	2012	‒	‒	1	0	1	Mediterranean & Eurasia*
*Thorneochloa*	*here*	‒	‒	1	0	1	Western North America
*Timouria*	1916	0	0	0	0	1	East Asia
*Trikeraia*	1954	‒	0	0	0	3	East Asia
× *Achnella*	1993	‒	1	0	‒	‒	North America
× *Eriosella*	*here*	‒	‒	1	0	1	North America

Thomasson (1978, 1980, 1981, 1982, 1985) was the first to document the phylogenetic importance of the lemma epidermal pattern among the Stipeae genera, and Barkworth and Everett (1988) used this information to delineate relationships. Romaschenko et al. (2008, 2011, 2012, 2013) mapped lemma patterns onto DNA-derived phylogenetic trees and found two major types (first-named, described, typified, and tested): 1) the saw-like pattern common in Stipeae and widespread among grasses outside of this tribe, characterized by having long fundamental cells 2× longer than wide with sinuate to lobate sidewalls and cork cells usually paired with silica bodies; and 2) maize-like pattern confined only to achnatheroid grasses within Stipeae, characterized by having thin-walled fundamental cells that are approximately equal in length and width to shorter than wide with mostly straight sidewalls, and silica bodies that are similar in shape and alternate regularly with fundamental cells. The saw-like pattern is found in *Aciachne* Benth., *Ampelodesmos*, *Anatherostipa* (Hack. ex Kuntze) Peñail., *Barkworthia*, *Hesperostipa*, *Lorenzochloa* Reeder & C. Reeder, *Macrochloa* Kunth, *Neotrinia*, *Ortachne* Nees, *Orthoraphium* Nees, *Oryzopsis*, *Patis*, *Piptatheropsis*, *Piptochaetium*, *Ptilagrostiella*, *Ptilagrostis*, *Stipa*, and *Trikeraia* Bor, while the maize-like pattern is found in *Achnatherum*, *Amelichloa*, *Anemanthele* Veldkamp, *Austrostipa* S.W.L. Jacobs & J. Everett, *Celtica* F.M. Vázquez & Barkworth, *Eriocoma*, *Jarava*, *Nassella*, *Oloptum*, *Pappostipa*, *Pseudoeriocoma*, *Thorneochloa*, and *Timouria* Roshev. (Romaschenko et al. 2012). The achnatheroid clade is a strongly-supported worldwide lineage (BS = 100, PP =1.00) defined by the maize-like lemma epidermal pattern (Romaschenko et al. 2012).

We follow the results previously presented in our molecular studies and provide overall morphological evidence for all genera recognized in this manuscript (Romaschenko et al. 2012, 2013, 2014; Valdés Reyna et al. 2013). We circumscribe genera based on shared morphological characteristics and apply the concept of monophyly as supported by recent molecular DNA-derived phylogenies. We think it is unwise to recognize paraphyletic genera portrayed as grades, e.g., *Stipellula* Röser & H.R. Hamasha (Hamasha et al. 2012). Therefore, we recognize *Stipellulacapensis* (Thunb.) Röser & H.R. Hamasha as the only species in this genus as originally described by Tzvelev (1974, 2012). One alternative classification for the Stipeae might be the recognition of a single genus, *Stipa*. However, we feel this would be inappropriate and not informative, as would the recognition of *Triticum* L. for all species within the Triticeae or *Poa* L. for all species within the family. As our title indicates, our key applies only to North American Stipeae. Our new classification presented here is globally coherent because our previous molecular studies are based on a worldwide comprehensive sample.

In this paper we propose a new classification of the North American Stipeae, include a key to the native and introduced genera (and Ampelodesmeae) found in Canada, United States of America, and Mexico, and provide descriptions, new combinations, and 10 illustrations for the species of *Barkworthia* Romasch., P.M. Peterson & Soreng, *Eriocoma*, *Neotrinia*, *Oloptum*, *Pseudoeriocoma* Romasch., P.M. Peterson & Soreng, *Ptilagrostiella* Romasch., P.M. Peterson & Soreng, *Thorneochloa* Romasch., P.M. Peterson & Soreng, and the hybrid genus ×*Eriosella* Romasch.

## Taxonomy

### Barkworthia

Taxon classificationPlantaePoalesPoaceae

Romasch., P.M.Peterson & Soreng, gen. nov.

urn:lsid:ipni.org:names:77199063-1

#### Type.

*Barkworthiastillmanii* (Bol.) Romasch., P.M. Peterson & Soreng (≡ *Stipastillmanii* Bol.).

#### Diagnosis.

*Barkworthia* differs from *Piptatherum* P. Beauv. in having spikelets with a pilose callus, paleas with prolonged veins, and 2-lobed lemma apices with lobes 1–3 mm long; and differs from *Achnatherum* in having saw-like lemma epidermal pattern, not the maize-like pattern characteristic of all achnatheroid grasses.

#### Description.

Plants short-rhizomatous perennials. Culms 60–150 cm tall with 2–5 puberulent nodes, 2–5 mm thick below, often geniculate. Leaf sheaths mostly glabrous or distally ciliate; collars glabrous or pubescent; ligules 0.2–0.5 mm long, membranous, apex truncate; blades 15–30 cm long; 3–7 mm wide, scabrous. Panicles 10–24 cm long, 1.5–3 cm wide, contracted; branches appressed, ascending, lower branches 2–3.5 cm long. Spikelets 14–18 mm long, lanceolate, subterete with one fertile floret without rachilla extension, disarticulation above the glumes; glumes 14–18 mm long, single-awned, the awns 2–3 mm long; lower glumes 1–3-veined, upper glumes 3–5-veined; florets 8–10 mm long, fusiform; calluses 0.5–1.2 mm long, rounded, pilose; lemmas 3-veined, evenly hairy, the hairs about 1.5 mm long, apex 2-lobed, the lobes 1–3 mm long with awnlike tips, narrow; lemma epidermal pattern saw-like; fundamental cells of variable length with sinuous sidewalls 2–7 times longer than silica cells irregularly alternating; silica bodies elongated-rectangular with straight or very shallow contracted sidewalls; cork cells not prominent; lemmatal awns 18–30 mm long, terminal, awned from the sinus, scabrous, 1 or 2-geniculate, persistent; paleas as long or longer than lemmas, 2-veined, hairy, the veins 1–3 mm prolonged reaching almost to the tip of the lemma lobes; anthers 4–6 mm long, penicillate, 3 in number; lodicules 3; stigmas 2. Caryopses fusiform, pericarp adherent, hilum linear.

#### Etymology.

The generic name honors Mary Elizabeth Barkworth, a well-known American agrostologist, who has contributed many papers investigating the taxonomy of the Stipeae.

### Barkworthia stillmanii

Taxon classificationPlantaePoalesPoaceae

(Bol.) Romasch., P.M.Peterson & Soreng, comb. nov.

urn:lsid:ipni.org:names:77199064-1

**Figure 1.**
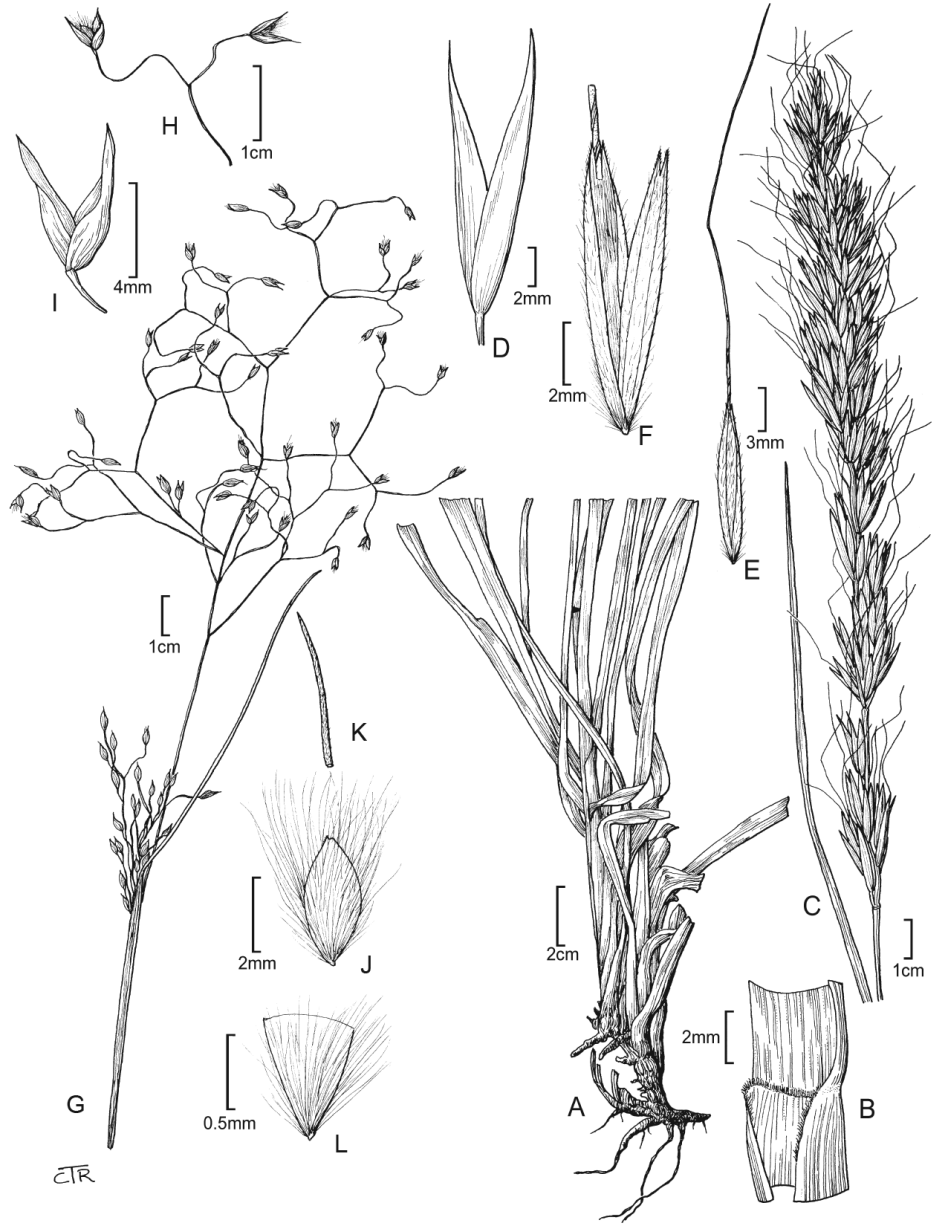
*Barkworthiastillmanii*: **A** habit **B** ligule **C** panicle **D** glumes **E** floret **F** floret (close up). *Eriocomahymenoides*: **G** culm and panicle **H** panicle branch **I** glumes **J** floret **K** lemma awn **L** floret base (callus).

Stipa stillmanii Bol., Proc. Calif. Acad. Sci. 4: 169. 1872 [Basionym] ≡ Achnatherumstillmanii (Bol.) Barkworth, Phytologia 74(1): 14. 1993 – Type: USA, California, Sierra Nevada, Blue Cañon, Jul 1870, *H.N. Bolander*, *M.D. Kellogg & co. s.n.* (holotype: NY-00431576 [image!]; isotypes: GH-00017890 [image!], K-000873398 [image!], MO-3055652!, MO-3055653!, MO-3055654!, NDG-07159 [image!], US-556922!). Fig. 1A–F.

#### Distribution and habitat.

*Barkworthiastillmanii* is distributed in scattered locations in northern California (Butte, El Dorado, Nevada, Placer, Plumas, Sacramento, Shasta, Sierra, Tehama, Trinity, Tulare, and Yuba Counties) associated with yellow pine and red fir forests; 10–1500 m (Barkworth 2007; Calflora 2018).

#### Comments.

In a molecular-derived phylogeny of the Stipeae using 10 DNA markers *Barkworthiastillmanii* is sister to a well-supported *Piptatherum* clade, which is strictly Old World in distribution, and has cauducous awns, and dark glossy lemmas in fruit (Romaschenko et al. 2012).

### Eriocoma

Taxon classificationPlantaePoalesPoaceae

Nutt., Gen. N. Amer. Pl. 1: 40. 1818

= Fendleria Steud., Syn. Pl. Glumac. 1: 419. 1854. Type: Fendleriarhynchelytroides Steud. (= Eriocomahymenoides).

#### Type.

*Eriocomahymenoides* (Roem. & Schult.) Rydb. (≡ *Stipahymenoides* Roem. & Schult.).

#### Description.

Plants perennial, sometimes short rhizomatous, tightly to loosely cespitose. Culms 10–230 cm tall, erect, unbranched above, nodes glabrous or pubescent, nodes 2–4 (5). Leaf sheaths glabrous, pubescent or pilose, glabrous or distally ciliate; collars glabrous or with a tufts of hairs; ligules 0.1–10 mm long, hyaline to membranous, apex truncate, obtuse, acute or narrowly acute; blades 0.1–7 mm wide, flat, convolute or involute, smooth, scabrous, glabrous or hairy. Panicles 2.5–60 cm long, up to 15 cm wide, usually contracted, sometimes open with divergent branches; branches straight, sometimes flexuous. Spikelets 5–21 mm long, usually lanceolate, sometimes obovoid, subterete, rarely laterally compressed, with one fertile floret without rachilla extension, disarticulation above the glumes; glumes 5–21 mm long, longer than the florets, unawned, 1(3)-veined, apex usually acuminate, sometimes acute; florets 2.5–10 mm long, usually fusiform, sometimes obovoid; calluses 0.3–2 mm long, blunt, sharp, or acute, hairy; lemmas usually coriaceous, sometimes indurate, usually evenly hairy, sometimes glabrous, or distally or with longer or shorter hairs than the body, apex usually entire or 2-lobed with lobes less than 2.1 mm long; lemma epidermal pattern maize-like; fundamental cells square with roundish corners and straight sidewalls subequal to silica cells or shorter, often regularly alternating; silica bodies square-cornered or sometimes rounded without contractions; cork cells scarce to absent; lemmatal awns 3–80 mm long, 1 or 2-geniculate; paleas ¼ to as long or longer than the lemma, 2-veined, usually hairy, sometimes glabrous, veins usually not prolonged, but if prolonged then not more than 0.3 mm long; anthers 1–5 mm long, usually penicillate, 3 in number; lodicules 2 or 3; stigmas 2. Caryopses fusiform, pericarp adherent, hilum linear.

#### Distribution.

There are 27 species of *Eriocoma*, all occurring in western North America (Canada, Mexico, and the USA) and only *E.hymenoides* extends its range into northeastern USA (Gleason and Cronquist 1991).

#### Comments.

Within our earlier and unpublished molecular analyses of *Eriocoma* there are three separate clades of *E.lobata* and one undescribed species (Romaschenko et al. 2012, 2014; Valdés Reyna et al. 2013; Romaschenko et al. in prep.). Species now included in *Eriocoma* were placed in *Oryzopsis* or *Stipa* (Hitchcock 1951), in *Stipa* (Espejo Serna et al. 2000), in *Achnatherum* (Barkworth 2007; Dávila et al. 2018), or in *Achnatherum* or *Eriocoma* (Sánchez-Ken 2018).

### Eriocoma alta

Taxon classificationPlantaePoalesPoaceae

(Swallen) Romasch., comb. nov.

urn:lsid:ipni.org:names:77199065-1

Stipa alta Swallen, Proc. Biol. Soc. Wash. 56: 79. 1943 [Basionym] ≡ Achnatherumaltum (Swallen) Hoge & Barkworth, Phytologia 74(1): 5. 1993. Type: Mexico, Coahuila, mpio. Cuatro Cienegas, Sierra de la Madera, Canon del Agua, rare in dry shrub zones of lower canyon, 10 Sep 1939, *C. H. Muller 3261* (holotype: US-2209361!; isotypes: GH-00024473 [image!], US-2871136!).

### Eriocoma arida

Taxon classificationPlantaePoalesPoaceae

(M.E. Jones) Romasch., comb. nov.

urn:lsid:ipni.org:names:77199066-1

Stipa arida M.E. Jones, Proc. Calif. Acad. Sci., ser. 2, 5: 725. 1895 [Basionym] ≡ Achnatherumaridum (M.E. Jones) Barkworth, Phytologia 74(1): 6. 1993. Type: USA, Utah, Piute Co., Marysvale, 6000 ft, 4 Jun 1894, *M.E. Jones 5377* (holotype: not located; isotypes: AHUC-13276 [image!], BM-001042155 [image!], G-00176508 [image!], MO-2151568 [image!], MSC-0092934 [image!], US-236787!).
= Stipamormonum Mez, Repert. Spec. Nov. Regni Veg. 17: 209. 1921. Type: USA, Utah, Milford, 21 Jun 1880, 5000 ft, *M.E. Jones 2106* (lectotype: MO-2151566 [image!] **designated here**; isolectotypes: S-G-5821 [image!], US-866079 fragm. ex B! [image 00157472!]).

### Eriocoma arnowiae

Taxon classificationPlantaePoalesPoaceae

(S.L. Welsh & N.D. Atwood) Romasch, comb. nov.

urn:lsid:ipni.org:names:77199067-1

Stipa arnowiae S.L. Welsh & N.D. Atwood, Utah Fl. (ed. 3) 799. 2003 [Basionym] ≡ Achnatherumarnowiae (S.L. Welsh & N.D. Atwood) Barkworth, Sida 22(1): 496. 2006. Type: USA, Utah, Kane Co., T43S, R4W, S13, ca. 9 mi E of Johnson Canyon Jct,, 1740 m, 30 May 2003, *S. L. Welsh & T. O’Dell 28062* (holotype: BRY; isotypes: GH-00247115 [image!], NY-00887984 [image!], US-3498681!).

### Eriocoma bloomeri

Taxon classificationPlantaePoalesPoaceae

(Bol.) Romasch. comb. nov.

urn:lsid:ipni.org:names:77199068-1

Stipa bloomeri Bol., Proc. Calif. Acad. Sci. 4: 168. 1872 [Basionym] ≡ Oryzopsisbloomeri (Bol.) Ricker, Contr. U.S. Natl. Herb. 11: 109. 1906 ≡ ×Stiporyzopsisbloomeri (Bol.) B.L. Johnson, Amer. J. Bot. 32: 602, f. 14–18. 1945 ≡ Achnatherum×bloomeri (Bol.) Barkworth, Phytologia 74(1): 14. 1993 Type: USA, California, Bloody Canyon near Mono Lake, Sep 1866, *H.N. Bolander 6116* (lectotype: US-2947421! [US-00141573 image!] **designated here**, partial lectotype [collection number only] designated by Hitchcock Flora N. Amer. 17(6): 429. 1935; isolectotypes: CAS-0005671 [image!], K-000912826 [image!], K-000912827 [image!], MO-2151483 [image!], MO-2151484[image!], UC-38998 [image!].

### Eriocoma bracteata

Taxon classificationPlantaePoalesPoaceae

(Swallen) Romasch., comb. nov.

urn:lsid:ipni.org:names:77199069-1

Stipa bracteata Swallen, J. Wash. Acad. Sci. 30(5): 213. 1940 [Basionym] ≡ Achnatherumbracteatum (Swallen) Valdés-Reyna & Barkworth, Contr. U.S. Natl. Herb. 48: 15. 2003. Type: Mexico, Baja California, collected on grassy flats 25 mi N of Ensenada, 4 Apr 1931, *I.L. Wiggins 5153* (holotype: US-1721797!; isotypes: CAS-0004680 [image!], CAS-0004681 [image!], GH-00024475 [image!].

### Eriocoma contracta

Taxon classificationPlantaePoalesPoaceae

(B.L. Johnson) Romasch., comb. nov.

urn:lsid:ipni.org:names:77199070-1

Oryzopsis hymenoides var. contracta B.L. Johnson, Bot. Gaz. 107: 24. 1945 [Basionym] ≡ Oryzopsiscontracta (B.L. Johnson) Y. Schechter, Brittonia 18: 342. 1967 ≡ Stipacontracta (B.L. Johnson) W.A. Weber, Phytologia 67(6): 428. 1989, nom. illeg. hom., non Stipacontracta Phil. ≡ Achnatherumcontractum (B.L. Johnson) Barkworth, Phytologia 74(1): 6. 1993. Type: USA, Wyoming, Carbon Co., Freezeout Hills, *E. Nelson 4850* (holotype: RM-0000328 [image!].

### Eriocoma coronata

Taxon classificationPlantaePoalesPoaceae

(Thurb.) Romasch., comb. nov.

urn:lsid:ipni.org:names:77199071-1

Stipa coronata Thurb., Bot. California 2: 287–288. 1880 [Basionym] ≡ Achnatherumcoronatum (Thurb.) Barkworth, Phytologia 74(1): 6. 1993. Type: USA, California, San Diego Co., in a cañon around springs on hillside near Julian City, Apr 1872, *H.N. Bolander*, *A. Kellogg & co. s.n.* (lectotype: US-745776 [accession no.!] & US-00406146 [image!] **designated here**; isolectotypes: GH-00017898 [image!], MO-2151562 [image!], MO-2151563 [image!], MO-2151564 [image!]).

### Eriocoma curvifolia

Taxon classificationPlantaePoalesPoaceae

(Swallen) Romasch., comb. nov.

urn:lsid:ipni.org:names:77199072-1

Stipa curvifolia Swallen, J. Wash. Acad. Sci. 23(10): 456. 1933 [Basionym] ≡ Achnatherumcurvifolium (Swallen) Barkworth, Phytologia 74(1): 7. 1993. Type: USA, New Mexico, Eddy Co., Guadalupe Mountains, in crevices of limestone cliff near mouth of North Fork of Rocky Arroyo, 29 Apr 1932, *H. Wilkens 1660* (holotype: US-1538063!; isotype: PH-00028074 [image!]).

### Eriocoma hendersonii

Taxon classificationPlantaePoalesPoaceae

(Vasey) Romasch., comb. nov.

urn:lsid:ipni.org:names:77199073-1

Oryzopsis hendersonii Vasey, Contr. U.S. Natl. Herb. 1(8): 267 [Basionym] ≡ Oryzopsisexiguavar.hendersonii (Vasey) M.E. Jones, Contr. W. Bot. 14: 11. 1912 ≡ Stipahendersonii (Vasey) Mehlenb., Canad. J. Bot. 49(9): 1568. 1971 ≡ Achnatherumhendersonii (Vasey) Barkworth, Phytologia 74(1): 7. 1993. 1893. Type: USA, Washington, North Yakima, Clements Mountain, 1892, *L.F. Henderson 2249* (holotype: US-81978!).

### Eriocoma hymenoides

Taxon classificationPlantaePoalesPoaceae

(Roem. & Schult.) Rydb., Bull. Torrey Bot. Club 39(3): 102. 1912.

Stipa hymenoides Roem. & Schult., Syst. Veg. 2: 339. 1817 [Basionym] ≡ Stipamembranacea Pursh, Fl. Amer. Sept. II: 728. 1814 nom. illeg., non Stipamembranacea L. ≡ Oryzopsismembranacea Vasey, U.S.D.A. Div. Bot. Bull. 12(2): 10, t. 10. 1891, nom. illeg. superfl. ≡ Eriocomamembranacea (Vasey) Beal, Grass. N. Amer. 2: 232. 1896, nom. illeg. superfl. ≡ Oryzopsishymenoides (Roem. & Schult.) Ricker ex Piper, Contr. U.S. Natl. Herb. 11: 109. 1906 ≡ Achnatherumhymenoides (Roem. & Schult.) Barkworth, Phytologia 74(1): 7–8. 1993. Type: USA, on the banks of the Missouri River, *J. Bradbury no. 12* (lectotype: K-000912825 [image!] **designated here**; isolectotype: PH-00008181 [image!]). Fig. 1G–L.
= Eriocomacuspidata Nutt., Gen. N. Amer. Pl. 1: 40. 1818 ≡ Miliumcuspidatum (Nutt.) Spreng., Syst. Veg. 1: 251. 1824 ≡ Urachnelanata Trin. & Rupr., Mem. Acad. Imp. Sci. Saint-Petersbourg, Ser. 6, Sci. Math., Seconde Pt. Sci. Nat. 3,1(2–3): 126. 1834, nom. Illeg. superfl. ≡ Eriocomamembranacea Steud., Nomencl. Bot. (ed 2) 1: 586. 1840, nom. inval., as syn. of Urachnelanata Trin. ≡ Oryzopsiscuspidata (Nutt.) Benth. ex Vasey, Grass. U.S. 23. 1883. Type: USA, Platte Plains, *T. Nuttall s.n.* (lectotype: BM-001042144 [image!]) **designated here**; isolectotype: LE-TRIN 1466.01 ex PH!).
= Fendleriarhynchelytroides Steud., Syn. Pl. Glumac. 1: 420. 1854. Type: USA, New Mexico, near Santa Fe, 1847, *A. Fendler 979* (lectotype: P-01941338 [image!] **designated here** ; isolectotypes: GH-00023719 [image!], K-000912824 [image!], NY [image!], S14-1154 [image!], US-823154 [image!], W-0029207 [image!], W-0029208 [image!], W-18890236595 [image!].

### Eriocoma latiglumis

Taxon classificationPlantaePoalesPoaceae

(Swallen) Romasch., comb. nov.

urn:lsid:ipni.org:names:77199074-1

Stipa latiglumis Swallen, J. Wash. Acad. Sci. 23(4): 198, f. 1. 1933 [Basionym] ≡ Achnatherumlatiglume (Swallen) Barkworth, Phytologia 74(1): 8. 1993. Type: USA, California, Yosemite Valley, Camp Lost Arrow, 4000–4500 ft, 22 Jun 1911, *L. Abrams 4469* (holotype: US-992334!; isotype: US-59760!).

### Eriocoma lemmonii

Taxon classificationPlantaePoalesPoaceae

(Vasey) Romasch., comb. nov.

urn:lsid:ipni.org:names:77199075-1

Stipa pringlei var. lemmonii Vasey, Contr. U.S. Natl. Herb. 3(1): 55. 1892 [Basionym] ≡ Stipalemmonii (Vasey) Scribn., Circ. Div. Agrostol. U.S.D.A. 30: 3. 1901 ≡ Achnatherumlemmonii (Vasey) Barkworth, Phytologia 74(1): 8. 1993. Type: USA, California, Plumas Co., Mohawk Valley, May 1889, *J.G. Lemmon 5456* (holotype: US-556900!).
= Stipacolumbiana Macoun, Cat. Canad. Pl. 2(4): 191. 1888, nom. utique rej. under International Code of Botanical Nomenclature (ICBN 1988) Art. 56.1, (see ICNAFP 2018 - Appendix V; also Barkworth and Maze 1979). Type: Canada, British Columbia, Yale, on rocks, 17 May 1875, *J. Macoun 28940* (lectotype: CAN-9899 designated by Hitchcock, Contr. U.S. Natl. Herb. 24(7): 253. 1925; isolectotype: US-77975!).

### Eriocoma lemmonii subsp. pubescens

Taxon classificationPlantaePoalesPoaceae

(Crampton) Romasch., comb. nov.

urn:lsid:ipni.org:names:77199076-1

Stipa lemmonii var. pubescens Crampton, Leafl. W. Bot. 7(9): 220. 1955 [Basionym] ≡ Achnatherumlemmoniisubsp.pubescens (Crampton) Barkworth, Phytologia 74(1): 8. 1993. Type: USA, California, Tehama Co., Whitlock Camp, Round Mt. area west of Paskenta, 4000 ft, 16 Jul 1954, *B. Crampton 2000* (holotype: AHUC-21077 [image!]; isotypes: AHUC-21078 [image!], CAS-0005669 [image!], US-2152024!).
= Stipalemmoniivar.jonesii Scribn., Bull. Div. Agrostol., U.S.D.A. 30: 4. 1901. Type: USA, California:, Emigrant Gap, 28 Jun 1882, *M.E. Jones 3298* (holotype: US-556899! [US-00141633 image!]; isotypes: BR-0000006884598 [image!], CAS-0005668 [image!], GH-00017900 [image!], MO-2151560 [image!], NY-00431560 [image!]), POM-116527).

### Eriocoma lettermanii

Taxon classificationPlantaePoalesPoaceae

(Vasey) Romasch., comb. nov.

urn:lsid:ipni.org:names:77199077-1

Stipa lettermanii Vasey, Bull. Torrey Bot. Club 13: 53. 1886 [Basionym] ≡ Stipaviridulavar.lettermanii (Vasey) Vasey, Contr. U.S. Natl. Herb. 3(1): 50. 1892 ≡ Achnatherumlettermanii (Vasey) Barkworth, Phytologia 74(1): 9. 1993. Type: USA, Idaho, Snake River, Aug 1885, *G.W. Letterman 102* (lectotype: US-556904! designated by Hitchcock, Manual. Grass. US ed. 1, 964. 1935 as to the collection no. *102*, Barkworth & Maze identifed the US specimen number, Taxon 31(2): 294 f. 6. 1982).
= Stipaviridulavar.minor Vasey, Contr. U.S. Natl. Herb. 3(1): 50. 1892 ≡ Stipaoccidentalisvar.minor (Vasey) C.L. Hitchc., Vasc. Pl. Pacific NW 1: 714. 1969 ≡ Stipaminor (Vasey) Scribn., Bull. Div. Agrostol., U.S.D.A. 11: 46–47. 1898. Type: USA, Colorado, Kelso Mountain near Torrey’s Peak, 13000 ft, 13 Aug 1885, *G.W. Letterman 95* (lectotype: US-556903! designated by Hitchcock, Contr. U.S. Natl. Herb. 24(7): 253. 1925).

### Eriocoma lobata

Taxon classificationPlantaePoalesPoaceae

(Swallen) Romasch. comb. nov.

urn:lsid:ipni.org:names:77199078-1

**Figure 2.**
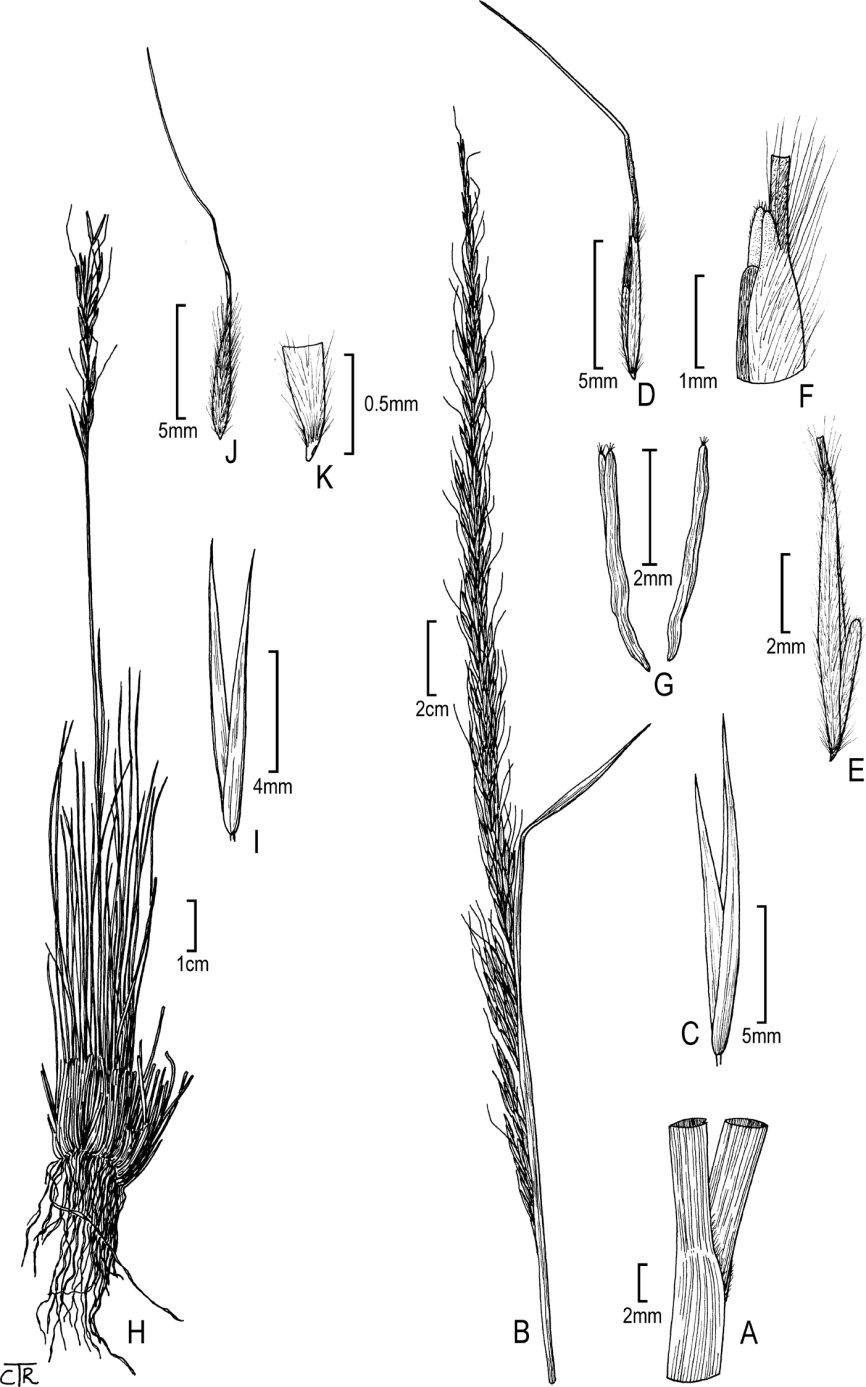
*Eriocomalobata*: **A** sheath and blade **B** panicle **C** glumes **D** floret **E** floret (close up) **F** lemma apex **G** anthers. *Eriocomapinetorum*: **H** habit **I** glumes **J** floret **K** floret base (callus).

Stipa lobata Swallen, J. Wash. Acad. Sci. 23(10): 199, f. 2. 1933 [Basionym] ≡ Achnatherumlobatum (Swallen) Barkworth, Phytologia 74(1): 9. 1993. Type: USA, New Mexico, Guadalupe Co., Queen, Guadalupe Mts., on a rocky hill, Ranger Station, 6000–7000 ft, 3–6 Sep 1915, *A. S. Hitchcock 13502* (holotype: US-905722!). Fig. 2A–G.

### Eriocoma nelsonii

Taxon classificationPlantaePoalesPoaceae

(Scribn.) Romasch., comb. nov.

urn:lsid:ipni.org:names:77199079-1

Stipa nelsonii Scribn., Bull. Div. Agrostol., U.S.D.A. 11: 46. 1898 [Basionym] ≡ Stipacolumbianavar.nelsonii (Scribn.) Hitchc., Contr. U.S. Natl. Herb. 24(7): 254. 1925 ≡ Stipacolumbianavar.nelsonii (Scribn.) H. St. John, Fl. S.-E. Washington 61. 1937 ≡ Stipaoccidentalisvar.nelsonii (Scribn.) C.L. Hitchc., Vasc. Pl. Pacific NW 1: 715. 1969 ≡ Achnatherumnelsonii (Scribn.) Barkworth, Phytologia 74(1): 9. 1993. Type: USA, Wyoming, Albany Co., Woods Landing, 2600 m, 9 Aug 1898, *A. Nelson 3963* (lectotype: US-556901! designated by Barkworth, Phytologia 74(1): 9. 1993; isolectotype: MPU-026968 [image!]).
= Stipawilliamsii Scribn., Bull. Div. Agrostol., U.S.D.A. 11: 45–46, t. 4. 1898. Type: USA, Wyoming, dry soil on W side of Big Horn Mt., near Monument Spring, 2200–2400 m, 3 Aug 1897, *T.A. Williams 2804* (lectotype: US-556907! & US-00141714 [image!] **designated here**, partially lectotypified by Hitchcock, N. Amer. Fl., part 6. 422. 1935).

### Eriocoma nelsonii subsp. dorei

Taxon classificationPlantaePoalesPoaceae

(Barkworth & J. Maze) Romasch., comb. nov.

urn:lsid:ipni.org:names:77199080-1

Stipa nelsonii subsp. dorei Barkworth & J. Maze, Taxon 28(5/6): 623 [Basionym] ≡ Stipanelsoniivar.dorei (Barkworth & J. Maze) Dorn, Vasc. Pl. Wyoming 298. 1988 ≡ Achnatherumnelsoniisubsp.dorei (Barkworth & J. Maze) Barkworth, Phytologia 74(1): 9. 1993. 1979. Type: Canada, Alberta, Dungarvan Creek, *W.G. Dore 12136* (holotype: DAO-000465415 [image!]).

### Eriocoma nevadensis

Taxon classificationPlantaePoalesPoaceae

(B.L. Johnson) Romasch., comb. nov.

urn:lsid:ipni.org:names:77199081-1

Stipa nevadensis B.L. Johnson, Amer. J. Bot. 49: 257. 1962 [Basionym] ≡ Achnatherumnevadense (B.L. Johnson) Barkworth, Phytologia 74(1): 9. 1993. Type: USA, California, Mono Co., Upper Twin Lake, near Bridgeport, 7096 ft, 29 Aug 1960, *B.L. Johnson 211* (holotype: UC-1936202 [image!]; isotypes: ARIZ-BOT-0005299[image!], DAV-181068 [image!], SD-00000116 [image!]).

### Eriocoma occidentalis

Taxon classificationPlantaePoalesPoaceae

(Thurb. ex S. Watson) Romasch., comb. nov.

urn:lsid:ipni.org:names:77199082-1

Stipa occidentalis Thurb. ex S. Watson, Botany (Fortieth Parallel) 380. 1871 [Basionym] ≡ Stipastrictavar.sparsiflora Vasey, Contr. U.S. Natl. Herb. 3(1): 51. 1892 ≡ Stipaoccidentalisvar.montana Merr. & Davy, Univ. Calif. Publ. Bot. 1: 62. 1902, nom. illeg. superfl. Achnatherumoccidentale (Thurb. ex S. Watson) Barkworth, Phytologia 74(1): 10. 1993 ≡ Type: USA, California, Yosemite Trail, 8000 ft, 20 Aug 1866, *H.N. Bolander 5038* (lectotype: GH-22338! designated by Hitchcock, Contr. U.S. Natl. Herb. 24(7): 242. 1925; isolectotypes: BM-000797606 [image], G-00176505 [image!], MO-2151636 [image!], NY-00431565 [image!], NY-00431567 [image!], US-3441781, US-992306 ex GH!, US-745821!, W-18890217496 [image!], YU-244757 [image!]).
= Stipastricta Vasey, Bull. Torrey Bot. Club 10: 42. 1883, nom. illeg. hom. non S.stricta Lam. ≡ Stipaoregonensis Scribn., Bull. Div. Agrostol., U.S.D.A. 17: 130, f. 426. 1899. Type: USA, “Oregon” [but from Washington, which became a state in 1889], 1882, *W.N. Suksdorf s.n.* (holotype: US-556921!).

### Eriocoma occidentalis subsp. californica

Taxon classificationPlantaePoalesPoaceae

(Merr. & Burtt Davy) Romasch., comb. nov.

urn:lsid:ipni.org:names:77199083-1

Stipa californica Merr. & Burtt Davy, Univ. Calif. Publ. Bot. 1: 61. 1902 [Basionym] ≡ Stipaoccidentalisvar.californica (Merr. & Burtt Davy) C.L. Hitchc., Vasc. Pl. Pacific NW 1: 715. 1969 ≡ Achnatherumoccidentalesubsp.californicum (Merr. & Burtt Davy) Barkworth, Phytologia 74(1): 10. 1993. Type: USA, California, San Jacinto Mts., north side of Fullers Ridge, 2100 m, Jul 1901, *H.M. Hall 2556* (holotype: unknown; isotypes: CAS-0005660 [image!], US-556911!).
= Stipanelsoniivar.longiaristata Barkworth & J. Maze, Taxon 28(5/6): 623. 1979 ≡ Achnatherumnelsoniisubsp.longiaristatum (Barkworth & J. Maze) Barkworth, Phytologia 74(1): 9. 1993. Type: USA, Washington, 8–9 mi W of Spokane, 19 Jun 1940, *J.S. Swallen 6231* (holotype: DAO-000465413 [image!]; isotype: US-2303647!).

### Eriocoma occidentalis subsp. pubescens

Taxon classificationPlantaePoalesPoaceae

(Vasey) Romasch., comb. nov.

urn:lsid:ipni.org:names:77199084-1

Stipa viridula var. pubescens Vasey, Contr. U.S. Natl. Herb. 3(1): 50. 1892 [Basionym] ≡ Stipaelmeri Piper & Brodie ex Scribn., Bull. Div. Agrostol., U.S.D.A. 11: 46. 1898 ≡ Stipaoccidentalisvar.pubescens (Vasey) J. Maze, Roy L. Taylor & MacBryde, Canad. J. Bot. 56(2): 193. 1978 ≡ Achnatherumoccidentalesubsp.pubescens (Vasey) Barkworth, Phytologia 74(1): 10. 1993. Type: USA, Washington, on dry ground along the Columbia River, 1883, *W.N. Suksdorf s.n.* (lectotype: US-79560! [US-00036944 image!], designated by Hitchcock, Contr. U.S. Natl. Herb. 24(7): 241. 1925; isolectotype: GH-00443467 [image!]).

### Eriocoma parishii

Taxon classificationPlantaePoalesPoaceae

(Vasey) Romasch., comb. nov.

urn:lsid:ipni.org:names:77199085-1

Stipa parishii Vasey, Bot. Gaz. 7(3): 33. 1882 [Basionym] ≡ Stipacoronatavar.parishii (Vasey) Hitchc., Contr. U.S. Natl. Herb. 24: 227, t. 50, f. 13. 1925 ≡ Achnatherumparishii (Vasey) Barkworth, Phytologia 74(1): 11. 1993. Type: USA, California, San Berardino Mts., Aug 1881, *S.B. Parish & W.F. Parish 1079* (lectotype: US-556918! & US-00406147 [image!] designated by Hitchcock, Contr. U.S. Natl. Herb. 24(7): 227. 1925).

### Eriocoma parishii subsp. depauperata

Taxon classificationPlantaePoalesPoaceae

(M.E. Jones) Romasch., comb. nov.

urn:lsid:ipni.org:names:77199086-1

Stipa parishii var. depauperata M.E. Jones, Contr. W. Bot. 14: 11. 1912 [Basionym] ≡ Stipacoronatavar.depauperata (M.E. Jones) Hitchc., J. Wash. Acad. Sci. 24(7): 292. 1934 ≡ Achnatherumparishiisubsp.depauperatum (M.E. Jones) Barkworth, Phytologia 74(1): 11. 1993. Type: USA, Utah, Detroit, 25 May 1891, *M.E. Jones s.n.* (holotype: RSA-0000500 [image!]; isotype: US-83026!).

### Eriocoma perplexa

Taxon classificationPlantaePoalesPoaceae

(Hoge & Barkworth) Romasch., comb. nov.

urn:lsid:ipni.org:names:77199087-1

Achnatherum perplexum Hoge & Barkworth, Phytologia 74(1): 11. 1993 [Basionym] ≡ Stipaperplexa (Hoge & Barkworth) Wipff & S.D. Jones, Phytologia 77(6): 461. 1995. Type: USA, New Mexico, Bernalillo Co., Cibola National Forest, 1.5 mi E of USFS road 413, 9 mi S of Tijeres on NM 14, 8 Sep 1985, *M.E. Barkworth 4764* (holotype: US-3239133!; isotype: RSA-0000391 [image!]).

### Eriocoma pinetorum

Taxon classificationPlantaePoalesPoaceae

(M.E. Jones) Romasch., comb. nov.

urn:lsid:ipni.org:names:77199088-1

Stipa pinetorum M.E. Jones, Proc. Calif. Acad. Sci., ser. 2, 5: 724. 1895 [Basionym] ≡ Achnatherumpinetorum (M.E. Jones) Barkworth, Phytologia 74(1): 12. 1993. Type: USA, Utah, Panguitch Lake, 8400 ft, growing in open places among the pine forests, 8 Sep 1894, *M.E. Jones 6023* (holotype: RSA-0000501 [image!]); isotype: US-236788!). Fig. 2H–K.

### Eriocoma richardsonii

Taxon classificationPlantaePoalesPoaceae

(Link) Romasch., comb. nov.

urn:lsid:ipni.org:names:77199089-1

Stipa richardsonii Link, Enum. Pl. 2: 245. 1833 [Basionym] ≡ Oryzopsisrichardsonii (Link) Beal, Bot. Gaz. 15(5)12: 111. 1890 ≡ Achnatherumrichardsonii (Link) Barkworth, Phytologia 74(1): 12. 1993. Type: Habitat in America boreali occidental, cultivated in Hortus Berolensis from seed sent by Richardson (lectotype: LE-TRIN-1436.01 fragm. ex B! **designated here**).

### Eriocoma robusta

Taxon classificationPlantaePoalesPoaceae

(Vasey) Romasch., comb. nov.

urn:lsid:ipni.org:names:77199090-1

**Figure 3.**
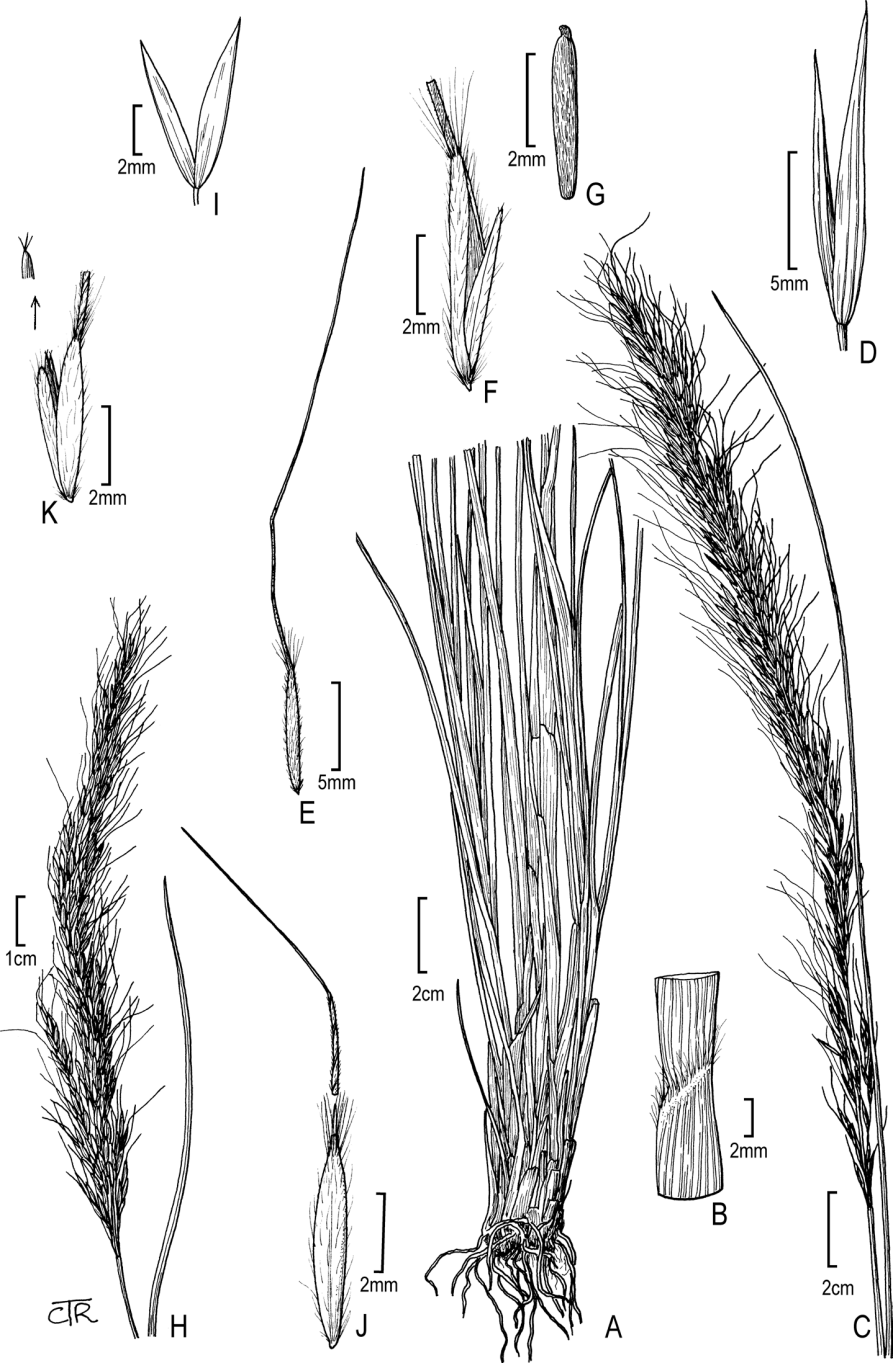
*Eriocomarobusta*: **A** habit **B** sheath and blade with a hairy collar **C** panicle **D** glumes **E** floret **F** floret (close up) **G** caryopsis. *Neotriniasplendens*: **H** panicle **I** glumes **J** floret **K** floret (close up) with anther tip (arrow).

Stipa viridula var. robusta Vasey, Contr. U.S. Natl. Herb. 1(2): 56. 1890 [Basionym] ≡ Stiparobusta (Vasey) Scribn., Bull. Div. Agrostol., U.S.D.A. 5: 23. 1897 ≡ Stipavaseyi Scribn., Bull. Div. Agrostol., U.S.D.A. 11: 46. 1898, nom. illeg. superfl. ≡ Achnatherumrobustum (Vasey) Barkworth, Phytologia 74(1): 12. 1993. Type: USA, New Mexico, 1881, *G.R. Vasey s.n.* (conserved type: US-993051!). Fig. 3A–G.

### Eriocoma scribneri

Taxon classificationPlantaePoalesPoaceae

(Vasey) Romasch., comb. nov.

urn:lsid:ipni.org:names:77199091-1

Stipa scribneri Vasey, Bull. Torrey Bot. Club 11: 125. 1884 [Basionym] ≡ Achnatherumscribneri (Vasey) Barkworth, Phytologia 74(1): 13. 1993. Type: USA, New Mexico, Santa Fe Co., Santa Fe, collected on dry hillsides, Aug 1884, *G.R. Vasey s.n.* (lectotype: US-556905! & US-00141676 [image!] designated by Barkworth in Phytologia 74(1): 13. 1993; isolectotypes: K-000873388 [image!], MO-2151550 [image!], MSC-0092941 [image!], NY-00431574 [image!], PH-00028089 [image!], US-84603!, W-19160026444 [image!]).

### Eriocoma swallenii

Taxon classificationPlantaePoalesPoaceae

(C.L. Hitchc. & Spellenb.) Romasch., comb. nov.

urn:lsid:ipni.org:names:77199092-1

Oryzopsis swallenii C.L. Hitchc. & Spellenb., Brittonia 20: 164. 1968 [Basionym] ≡ Achnatherumswallenii (C.L. Hitchc. & Spellenb.) Barkworth, Phytologia 74(1): 14. 1993. Type: USA, Idaho, Clark Co., just N of Birch Creek, along Hwy. 28, near the Lemhi Co. line, 7 Jul 1965, *C.L. Hitchcock 23868* (holotype: WTU-227273 & WTU-V-000041[image!]; isotypes: CAS-0006990 [image!], COLO-00391284 [image!], DAV-38298 [image!], DAO-000465414 [image!], F-0046857F [image!], G-00176562 [image!], GH-00024084 [image!], ID-00157718 [image!], NCU-00000362 [image!], NY-00381560 [image!], OSC-0001820 [image!], RM-0000329 [image!], RSA-0000458 [image!], TEX-00370123 [image!], UBC-V116845 [image!], US-3465271!, V-047552 [image!]).

### Eriocoma thurberiana

Taxon classificationPlantaePoalesPoaceae

(Piper) Romasch., comb. nov.

urn:lsid:ipni.org:names:77199093-1

Stipa thurberiana Piper, Circ. Div. Agrostol. U.S.D.A. 27: 10. 1900, nom. nov [Basionym] ≡ Stipaoccidentalis Thurb., U.S. Expl. Exped. 17: 483. 1874 non. Stipaoccidentalis Thurb. ex S. Watson ≡ Achnatherumthurberianum (Piper) Barkworth, Phytologia 74(1): 14. 1993. Type: USA, Washington, North Branch of the Columbia River, *C. Pickering & W. D. Brackenridge s.n.* (holotype: GH-00017772 [image!]).

### Eriocoma wallowaensis

Taxon classificationPlantaePoalesPoaceae

(J. Maze & K. Robson) Romasch., comb. nov.

urn:lsid:ipni.org:names:77199094-1

Achnatherum wallowaense J. Maze & K. Robson, Madrono 43(3): 401, f. 1–2. 1996 [Basionym]. Type: USA, Oregon, Wallowa Co., Wallowa-Whitman National Forest, ca. 34 km N of Enterprise, near Boner Gulch along Forest Service road, 46, 45°43'41.16"N, 117°08'10.32"W (SW 1/4 of SE 1/4, sect 24, T3N, R45E), 1481 m, 26 Jun 1993, *J. Maze*, *E. Maze*, *K.A. Robson & T. Henn 1007* (holotype: US-3323518!; isotypes: COLO-00339663 [image!], DAV-126946 [image!], DAV-128405 [image!], ID-00157713 [image!], MO-128384 [image!], NCU-00012752 [image!], NY-00039022 [image!], UBC-V209875 [image!]).

### Eriocoma webberi

Taxon classificationPlantaePoalesPoaceae

Thurb., Bot. California 2: 283–284. 1880

Eriocoma webberi Thurb., Bot. California 2: 283–284. 1880 [Basionym] ≡ Oryzopsiswebberi (Thurb.) Benth. ex Vasey, Grass. U.S. 23. 1883 ≡ Stipawebberi (Thurb.) B.L. Johnson, Bot. Gaz. 107: 25. 1945 ≡ Achnatherumwebberi (Thurb.) Barkworth, Phytologia 74(1): 14. 1993. Type: USA, California, Sierra Valley, 1 May 1871, *H.N. Bolander*, *A. Kellogg & co. s.n*. (holotype: GH-00024083 [image!]; isotypes: MO-2151485[image!], NY-00381032 [image!], US-81935!).

### × Eriosella

Taxon classificationPlantaePoalesPoaceae

Romasch., nothogen. nov.

urn:lsid:ipni.org:names:77199095-1

Eriocoma Nutt. × Nassella (Trin.) E. Desv. Type: ×Eriosellacaduca (Beal) Romasch. (≡ Oryzopsiscaduca Beal)

#### Description.

Plants perennial, cespitose, not rhizomatous. Culms up to 90 cm tall, nodes glabrous. Leaf sheaths mostly glabrous, margins sparsely ciliate, hairs longer apically; collars glabrous or with tufts of hairs; ligules 0.5–1.7 mm long, scarious, glabrous, apex truncate to obtuse; blades 1–3.5 mm wide, flat to convolute when dry, apices narrowly acute; basal blades to 40 cm long; flag blades longer than 10 cm. Panicles 15–18 cm long, narrow, branches ascending. Spikelets 6–8.5 mm long, fusiform, with one fertile floret without rachilla extension, disarticulation above the glumes; glumes 6–8.5 mm long, longer than the florets, saccate-lanceolate, 3–5-veined, apices attenuate from about the middle; upper glumes slightly narrower than the lower; florets 4–5 mm long, fusiform; calluses about 0.7 mm long, blunt; lemmas 7-veined, coriaceous, evenly hairy throughout, the hairs 1–2 mm long, apex minutely lobed; lemmatal awns 9–16 mm long, twisted, straight or 1-geniculate, readily deciduous, lower portion scabrous and without hairs; paleas 2.5–3.3 mm long, 2/3–3/4 as long as the lemma, hairy; stamens 2, anthers 1.2–2.3 mm long, variable in length within the floret, 2 in number indehiscent, penicillate, with only a few apical hairs. Caryopses not seen.

#### Etymology.

The name, ×*Eriosella*, is a combination of the prefix ‘Erio’ from *Eriocoma* and the suffix ‘*sella*’ from *Nassella*.

#### Distribution.

Known only from Montana, North Dakota, and western Wyoming (Johnson and Rogler 1943; Barkworth 2007).

### × Eriosella caduca

Taxon classificationPlantaePoalesPoaceae

(Beal) Romasch., comb. nov.

urn:lsid:ipni.org:names:77199096-1

Oryzopsis caduca Beal, Bot. Gaz. 15(5): 111. 1890 [Basionym] ≡ Eriocomacaduca (Beal) Rydb., Mem. New York Bot. Gard. 1: 25. 1900 ≡ Stipacaduca (Beal) Scribn., Contr. U.S. Natl. Herb. 3(1): 54. 1892 ≡ ×Stiporyzopsiscaduca (Beal) B.L. Johnson & Rogler, Amer. J. Bot. 30: 55, f. 10, 14, 28–33. 1943 ≡ ×Achnellacaduca (Beal) Barkworth, Phytologia 74(1): 15. 1993. Type: USA, Montana, Belt Mts., Sixteen Mile Cr., 11 Jul 1883, *F.L. Scribner s.n.* (holotype: US-745838!).

#### Comments.

×*Eriosellacaduca* is thought to be a hybrid between *Eriocomahymenoides* and *Nassellaviridula*. It can be separated from *E.hymenoides* in having shorter hairs on the lemma and panicles with ascending branches (not divergent), and from *N.viridula* in having longer lemma hairs, paleas 2/3–3/4 as long as the lemma, and readily deciduous lemmatal awns (Barkworth 2007). Another species similar to ×*Eriosellacaduca* is *Eriocomabloomeri*. However, the latter species has glabrous sheaths, shorter ligules, 5-veined lemmas, awns with a sub-plumose lower section below the bend, and anthers with more numerous apical hairs (Johnson and Rogler 1943).

### Neotrinia

Taxon classificationPlantaePoalesPoaceae

(Tzvelev) M. Nobis, P. Gudkova & A. Nowak, Turczaninowia 22 (1): 40. 2019

Achnatherum sect. Neotrinia Tzvelev, Novosti Sist. Vyssh. Rast. 9: 55. 1972.

#### Type.

*Neotriniasplendens* (Trin.) M. Nobis, P. Gudkova & A. Nowak (≡ *Stipasplendens* Trin.).

#### Description.

Plants perennial, cespitose, robust, not rhizomatous with intravaginal branching. Culms 40–250 cm tall, 2–5 mm thick below with 3–7 nodes, glabrous, smooth. Leaf sheaths glabrous, becoming fibrous below, margins ciliate, striate; collars glabrous; ligules membranous, glabrous; basal ligules 1–2.5 mm long, apex truncate to obtuse; upper ligules 2.5–12 mm long, apex acute; blades 20–60 cm long, 2–7 (–10) mm wide, flat or involute, deeply grooved, glabrous, abaxial surface smooth, adaxial surface scabrous. Panicles 15–50 cm long, (4–) 8–35 cm wide, ovate; ascending branches up to 15 cm long, crowded or loosely spreading, whorled at most nodes. Spikelets 4–7 (–8.5) mm long, lanceolate, subterete with one fertile floret without rachilla extension; disarticulation above the glumes; glumes 2.5–6.5 mm long, subequal, membranous, (1–) 3–5-veined, without keels; lower glumes 2.5–4.4 mm long, shorter than the upper, 1 (–3)-veined, margins hyaline; upper glumes 4–6.5 mm long, 3–5-veined, apex acute; florets 4.2–7.2 mm long; calluses 0.3–0.5 mm long, elliptic, bearded; lemmas 4.2–7.2 mm long, evenly hairy, the hairs up to 1.5 mm long, apex 2-lobed, the lobes 0.5–1.3 mm long; lemma epidermal pattern saw-like; fundamental cells of variable length with lobate sidewalls 3–10 times longer than silica cells, irregularly alternating; silica bodies round, paired with crescent-shaped cork cells; lemmatal awns 5–12 mm long, straight or indistinctly 1-geniculate, slightly twisted and flexuous; paleas about as long or slightly shorter than the lemmas, 2-veined, hairy; stamens 3, anthers 3.5–4.5 mm long, penicillate, yellow; lodicules 3; stigmas 2; ovary glabrous. Caryopses 2–4 mm long, fusiform, pericarp adherent, hilum linear. Chromosome number 2*n* = 42, 46, 48 (Freitag 1985; Gohil and Koul 1986).

### Neotrinia splendens

Taxon classificationPlantaePoalesPoaceae

(Trin.) M. Nobis, P. Gudkova & A. Nowak, Turczaninowia 22 (1): 40. 2019

Stipa splendens Trin., Neue Entdeck. Pflanzenk. 2: 54. 1821 [Basionym] ≡ Agrostislongiaristata Herb. in Ross. ex Kunth, Enum. Pl. 1: 178. 1833, nom. illeg. ≡ Lasiagrostissplendens (Trin.) Kunth, Révis. Gramin. 1: 58. 1829 ≡ Achnatherumsplendens (Trin.) Nevski, Trudy Bot. Inst. Akad. Nauk S.S.S.R., Ser. 1, Fl. Sist. Vyssh. Rast. 4: 224. 1937. Type: Russia, Transbaicalia, Siberia, *Fischer et Steven s.n.* (holotype: LE-TRIN1444.1!). Fig. 3H–K.

#### Distribution and habitat.

The single species, *Neotriniasplendens*, is native to Asia in Afghanistan, India, Kazakhstan, Kyrgyzstan, China, Mongolia, Pakistan, Russia, Tajikistan, Turkmenistan, and Uzbekistan (Wu and Phillips 2006). In North America *N.splendens* has been introduced as an ornamental (Barkworth 2007). The species occurs in cold, semi desert regions along drainages at 2100–3800 m (Freitag 1985).

#### Comments.

*Psammochloavillosa* (Trin.) Bor is sister to *Neotriniasplendens* in our earlier molecular phylogeny (BS = 100, PP = 1.00) and both species share the following morphological features: basal fibrous sheaths, panicles with whorled primary branches arising from the rachis, (1–) 3–5-veined glumes with hyaline margins, short, obtuse to elliptic calluses, and evenly hairy lemmas with flexuous cauducous awns that arise between the apical teeth (Freitag 1985; Wu and Phillips 2006; Barkworth 2007; Romaschenko et al. 2012). *Neotriniasplendens* differs from *Psammochloavillosa* in having cespitose culms without rhizomes, 4–7 (–8.5) mm long spikelets, 2.5–6.5 mm long glumes, 4.2–7.2 mm long lemmas that are evenly hairy with hairs up to 1.5 mm long, linear-lanceolate lodicules (versus flabellate), and 3.5–4.5 mm long anthers. It also differs from *Achnatherum* in having saw-like lemma epidermal pattern.

As noted by Barkworth (2007), the plants are rarely grazed upon, and sometimes form dense tall stands in Asia (RJS pers. obs.; Tzvelev 1976, p. 564 comment). *Achnatherumcaragana* (Trin.) Nevski was treated as the only other A.sect.Neotrinia species (Tzvelev 1976), and may belong to the genus.

### Oloptum

Taxon classificationPlantaePoalesPoaceae

Röser & H.R. Hamasha, Pl. Syst. Evol. 298: 365. 2012

#### Type.

*Oloptummiliaceum* (L.) Röser & H.R. Hamasha (≡ *Agrostismiliacea* L.).

#### Description.

Plants perennial, loosely cespitose, not rhizomatous with extravaginal branching. Culms 50‒150 cm tall, erect or geniculate ascending, glabrous, often branching at lower cauline nodes. Leaf sheaths glabrous, persistent, margins hyaline above, smooth; ligules membranous; basal ligules 0.5‒1.5 mm long, apex truncate; upper ligules 1.5‒4 mm long, apex obtuse to acute; blades (5‒) 10‒30 cm long, 2‒10 mm wide, flat, glabrous, smooth or scaberulous, margins scaberulous, apex attenuate. Panicles 10‒40 cm long, 3‒15 (‒18) cm wide, ovate, open; lower branches 3‒8 cm long, ascending and spreading, whorled, 3‒8 at a node or with 15‒30 or more at the lowest node, these often with sterile spikelets. Spikelets 2.5‒3.5 mm long, elliptic, dorsally compressed with one fertile floret without rachilla extension; disarticulation above the glumes; glumes subequal, longer than the florets, 3-veined without transverse veinlets, membranous, apices acuminate; florets 2‒2.5 mm long, chartaceous; calluses about 0.3 mm long, with non-grooved circular disarticulation scar, glabrous; lemmas with narrow open borders, central vein not grooved, apex awned, the awns 3‒5 mm long, flexuous, cauducous; lemma epidermal pattern maize-like; fundamental cells elongated with straight thin sidewalls 3–7 times longer than silica cells, irregularly alternating; silica bodies round; cork cells crescent-shaped scarce to absent; paleas about as long as the lemma, coriaceous, 2-veined; stamens 3, anthers 2‒2.5 mm long, penicillate; lodicules 3; stigmas 2; ovary glabrous. Caryopses 1.5‒1.7 mm long, fusiform, pericarp adherent, hilum linear about ½ as long as the caryopsis. Chromosome number 2*n* = 24 (Faruqi et al. 1987; Devesa et al. 1991; Luque and Lifante 1991; Verlaque et al. 1997).

### Oloptum miliaceum

Taxon classificationPlantaePoalesPoaceae

(L.) Röser & H.R. Hamasha, Pl. Syst. Evol. 298(365): 2012

**Figure 4.**
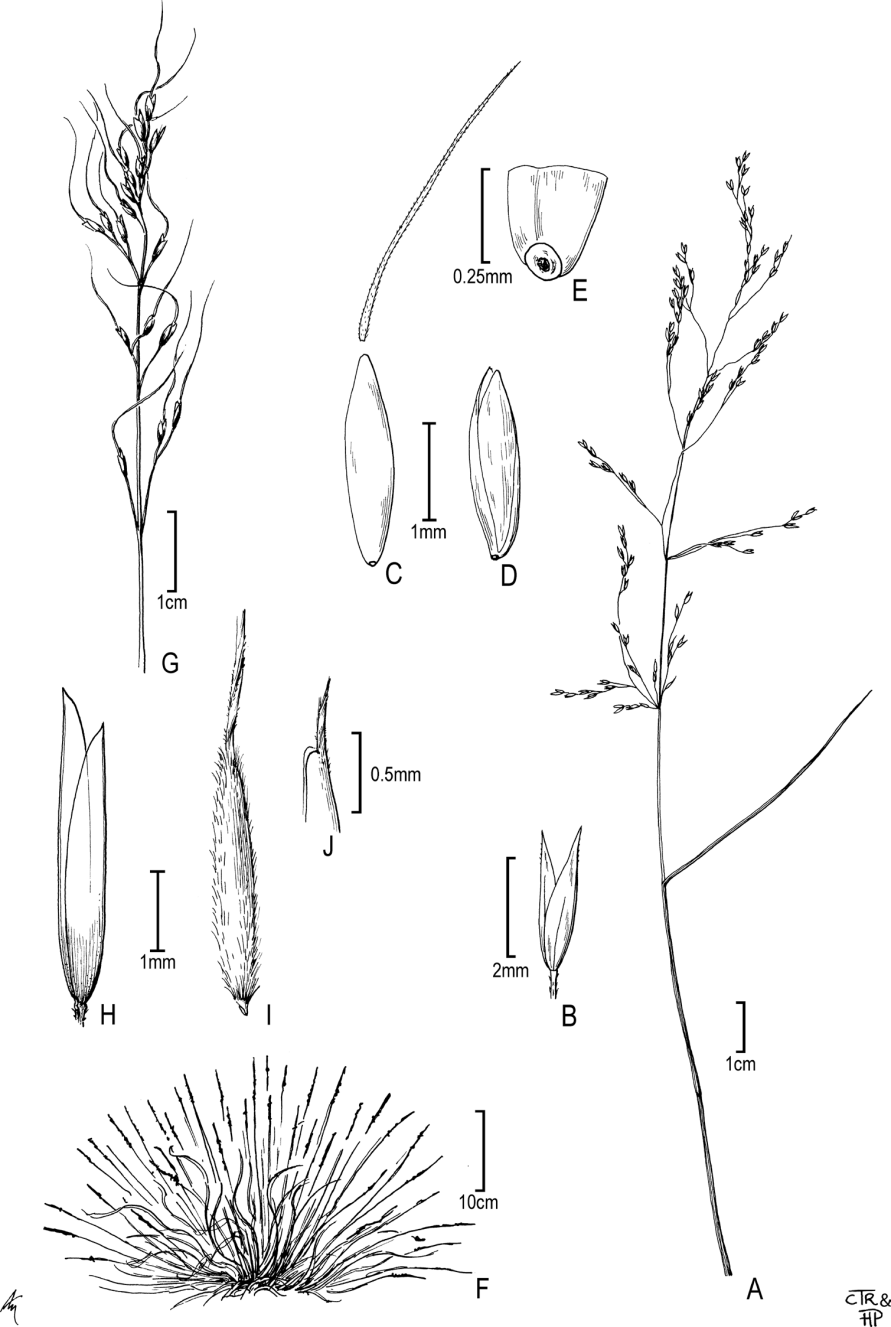
*Oloptummiliaceum*: **A** panicle **B** glumes **C** floret **D** lemma and palea **E** lemma base with disarticulation scar. *Ptilagrostiellakingii*: **F** habit **G** panicle **H** glumes **I** floret **J** lemma apex.

Agrostis mileacea L., Sp. Pl. 1: 61. 1753 [Basionym] ≡ Achnatherummiliaceum (L.) P. Beauv., Ess. Agrostogr. 20, 146, 148. 1812 ≡ Urachnemiliacea (L.) K. Koch, Linnaea 21(4): 439. 1848 ≡ Piptatherummiliaceum (L.) Coss., Notes Pl. Crit. 129. 1851 ≡ Oryzopsismiliacea (L.) Benth. & Hook. ex Asch. & Schweinf., Mém. Inst. Égypte 2: 169. 1887 ≡ Stipamiliacea (L.) Hoover, Leafl. W. Bot. 10(16): 340. 1966. Type: Sweden, Uppsala, *Anon. s.n.* (lectotype: LINN-HL84-2 [image!] designated by R.D. Meikle, Fl. Cyprus 2: 1794. 1985). Fig. 4A–E.

#### Distribution and habitat.

*Oloptummiliaceum* is native to Europe, particularly the whole Mediterranean region, from northern Africa, Sinai to Western Asia (Arabian Peninsula, Cyprus, Egypt, Iraq, Iran, Israel, Jordan, Lebanon, Palestinian territories, Syria, and Turkey) [Freitag 1975; Soreng et al. 2003; Ibrahim et al. 2016]. It is naturalized in southern Africa, Australia, New Zealand, North America (Arizona, California, Maryland) and South America (Barkworth 2007), and has been cultivated in Mississippi, North Carolina, Tennessee, and Utah (see SEINet http://swbiodiversity.org/seinet/collections/list.php?db=all&taxa=Achnatherum+miliaceum&usethes=1&taxontype=2&page=1). The species occurs in various disturbed habitats along roadsides, ditches, borders of fields, dry river beds, and dumping grounds usually below 2000 m (Freitag 1975).

#### Comments.

The unique morphological features of this taxon (glabrous lemma with a central vein not grooved, 3-veined glumes without transverse veinlets, and a callus with a circular disarticulation scar) were first recognized by Roshevitz (1951) and later officially named by Freitag (1975) as Piptatherumsect.Miliacea Roshev. ex Freitag. Lemma epidermal pattern of *Oloptum* is unusual among achnatheroid grasses resembling only that of *Celtica*. It is distinguished by having long fundamental cells irregularly alternating with silica bodies. In our earlier molecular analysis, *O.miliaceum* is sister to the Eurasian *Achnatherum* clade in the core *Achnatherum* clade, which also includes *Stipellula*, *Austrostipa* S.W.L. Jacobs & J. Everett, *Anemanthele* Veldkamp and *Celtica* F.M. Vázquez & Barkworth (Romaschenko et al. 2011, 2012).

Traditionally, two subspecies have been recognized. Oloptummiliaceumsubsp.thomasii (Duby) Boiss. differs from the typical form in having densely verticillate panicles with 15‒30 or more often sterile branches on the lowest whorl (Freitag 1975). There is genetic variation between these two subspecies in our earlier analyses (Romaschenko et al. 2011, 2012). A molecular study with a larger sample of the subspecies is necessary to fully explore evolutionary relationships.

### Pseudoeriocoma

Taxon classificationPlantaePoalesPoaceae

Romasch., P.M.Peterson & Soreng, gen. nov.

urn:lsid:ipni.org:names:77199097-1

#### Type.

*Pseudoeriocomaeminens* (Cav.) Romasch. (≡ *Stipaeminens* Cav.)

#### Diagnosis.

*Pseudoeriocoma* differs from *Eriocoma* Nutt. in having bamboo-like culms commonly with up to 13 nodes, 3–6 mm thick below, with ramified branching at the middle and upper nodes.

#### Description.

Plants perennial, cespitose, usually short rhizomatous from a knotty base. Culms 30–230 (often over 100) cm tall, erect or ascending, often geniculate, 3–6 mm thick and often woody and bamboo-like below with ramified and branching at the middle and upper nodes, with (2) 3–13 nodes, internodes glabrous or hairy. Leaf sheaths shorter than the internodes above to shorter or longer below, glabrous, pubescent or hirsute, sometimes ciliate on the margins and summit; collars glabrous or with a tuft of hairs; ligules 0.5–8 mm long, hyaline to membranous, apex truncate to acute or obtuse, often lacerate; blades (1.5–) 5–40 cm long, 1–4 mm wide, flat to tightly involute or convolute, glabrous or pubescent, usually scabrous. Panicles 8–45 (–55) cm long, usually rather narrow and less than 8.5 cm wide, loosely or densely flowered, branches ascending to spreading and naked near base; pedicles longer than the spikelets. Spikelets 8–15 mm long, lanceolate with one fertile floret without rachilla extension; disarticulation above the glumes; glumes (4–) 6–15 mm long, longer than the florets, subequal or unequal, hyaline to membranous, 1–7-veined, glabrous, acuminate; florets 4–7 mm long, usually fusiform; calluses 0.2–2 long, sharp, hairy; lemmas 4–7 mm long, fusiform, coriaceous, evenly hairy, the hairs 0.4–2 mm long, margins enveloping most of the palea, apex entire and awned; lemma epidermal pattern maize-like; fundamental cells squared, longitudinally compressed with straight thin sidewalls subequal to silica cells (silica bodies) or shorter, regularly alternating; cork cells absent; lemmatal awns 20–80 mm long, 2-geniculate, flexuous, the segments scabrous or pubescent; paleas 1–4.6 mm long, 1/3 to ¾ as long as the lemmas, 2-veined, veins not prolonged, hairy; anthers 2.5–4 mm long, penicillate or not, 3 in number, lodicules 2 or 3; stigmas 2. Caryopses 3–4 mm long, fusiform, pericarp adherent, hilum linear.

#### Distribution and habitat.

There are six species of *Pseudoeriocoma* occurring in southwestern North America (Mexico and USA). These species generally occur on steep rock outcrops in xerophytic vegetation; pinyon, pine, pine-oak woodlands, and spruce-fir forests; 600–3000 m (Barkworth 2007; Valdés Reyna 2015).

#### Comments.

Within our preliminary molecular analyses of *Pseudoeriocoma* there are two clades each of *P.constricta*, *P.eminens*, and *P.multinodis* that require further study, and at least three species currently placed in *Jarava* from South America that align within *Pseudoeriocoma* (Romaschenko et al. 2012, 2014; Valdés Reyna et al. 2013; Romaschenko et al. in prep.).

### Pseudoeriocoma acuta

Taxon classificationPlantaePoalesPoaceae

(Swallen) Romasch., comb. nov.

urn:lsid:ipni.org:names:77199098-1

Stipa acuta Swallen, J. Wash. Acad. Sci. 30(5): 212. 1940 [Basionym] ≡ Achnatherumacutum (Swallen) Valdés-Reyna & Barkworth, Contr. U.S. Natl. Herb. 48: 15. 2003. Type: Mexico, Coahuila, on rocky soil on Carneras Pass, 21 mi S of Saltillo, 1 Sep 1938, *F. Shreve 8545* (holotype: US-1760238!; isotype: ARIZ-BOT-0004856 [image!]).

### Pseudoeriocoma constricta

Taxon classificationPlantaePoalesPoaceae

(Hitchc.) Romasch., comb. nov.

urn:lsid:ipni.org:names:77199099-1

Stipa constricta Hitchc., Contr. U.S. Natl. Herb. 24(7): 244, t. 51, f. 28–29. 1925 [Basionym] ≡ Achnatherumconstrictum (Hitchc.) Valdés-Reyna & Barkworth, Contr. U.S. Natl. Herb. 48: 15. 2003. Type: Mexico, Hidalgo, Pachuca, collected on a rocky hill at 2400 m alt., 7 Sep 1910, *A.S. Hitchcock 6742* (holotype: US-993345!; isotype: NY-00431580 [image!]).

### Pseudoeriocoma editorum

Taxon classificationPlantaePoalesPoaceae

(E. Fourn.) Romasch., comb. nov.

urn:lsid:ipni.org:names:77199100-1

Stipa editorum E. Fourn., Mexic. Pl. 2: 75. 1886 [Basionym] ≡ Achnatherumeditorum (E. Fourn.) Valdés-Reyna & Barkworth, Contr. U.S. Natl. Herb. 48: 16. 2003. Type: Mexico, in valle edita inter La Noria del Viejo et La Miquiguana, *W.F. von Karwinski 1009c* (holotype: P; isotypes: KFTA-0002846 [image!], US-866119A! fragm. ex P).

### Pseudoeriocoma eminens

Taxon classificationPlantaePoalesPoaceae

(Cav.) Romasch., comb. nov.

urn:lsid:ipni.org:names:77199101-1

**Figure 5.**
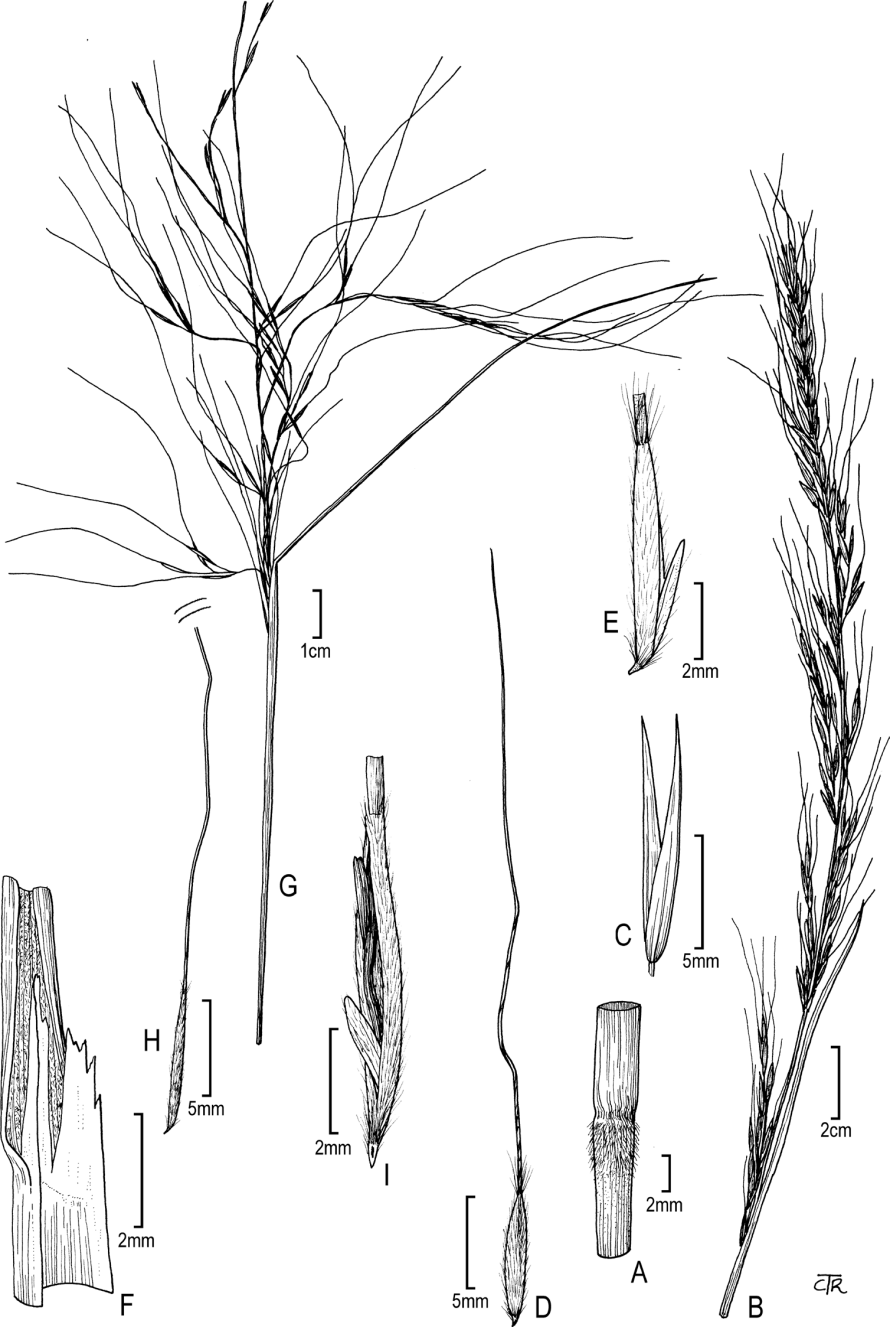
*Thorneochloadiegoensis*: **A** lower culm node **B** panicle **C** glumes **D** floret **E** floret (close up). *Pseudoeriocomaeminens*: **F** ligule **G** panicle **H** floret **I** floret (close up).

Stipa eminens Cav., Icon. 5: 42, t. 467, f. 1. 1799 [Basionym] ≡ Achnatherumeminens (Cav.) Barkworth, Phytologia 74(1): 7. 1993. Type: Mexico, Chalma, *L. Née s.n.* (holotype: MA-656523: isotype: US-866118!).
= Stipaerecta E. Fourn., Mexic. Pl. 2: 75. 1886, nom. illeg. hom., non Stipaerecta Trin. ≡ Stipaerecta E. Fourn., Biol. Cent.-Amer., Bot. 3: 536. 1885. nom. nud. Type: Mexico, Tehuacán, Dec, *F.M. Liebmann 654* (holotype: C-10017241 [image!]; isotype: US-866117! fragm. ex C).
= Stipaflexuosa Vasey, Bull. Torrey Bot. Club 15: 49. 1888. Type: USA, western Texas, Chenate Mountains, 1887, *G.C. Nealley s.n*. (holotype: US-556913!; isotypes: NY-00431557 [image!], W-19160022725 [image!]). Fig. 5F–I.

### Pseudoeriocoma hirticulmis

Taxon classificationPlantaePoalesPoaceae

(S.L. Hatch, Valdés-Reyna & Morden) Romasch., comb. nov.

urn:lsid:ipni.org:names:77199102-1

Stipa hirticulmis S.L. Hatch, Valdés-Reyna & Morden, Syst. Bot. 11(1): 186–188, f. 1. 1986 [Basionym] ≡ Achnatherumhirticulme (S.L. Hatch, Valdés-Reyna & Morden) Valdés-Reyna & Barkworth, Contr. U.S. Natl. Herb. 48: 16. 2003. Type: Mexico, Nuevo León, 8 mi E of San Roberto Jct. along Hwy. 58 on the road to Galeana, 24°40'N, 100°14'W, 1890 m, 22 Aug 1983, *S. Hatch & J. Valdés Reyna 5007* (holotype: TAES; isotypes: ANSM-028729 [image!], CHAPA-0000220 [image!], ENCB-003270 [image!], MEXU-00415572 [image!], MO-123113 [image!], NY-00431581 [image!], TEX-00370148 [image!], US-3037668!).

### Pseudoeriocoma multinodis

Taxon classificationPlantaePoalesPoaceae

(Scribn. ex Beal) Romasch., comb. nov.

urn:lsid:ipni.org:names:77199103-1

Stipa multinodis Scribn. ex Beal, Grass. N. Amer. 2: 222. 1896 [Basionym] ≡ Achnatherummultinode (Scribn. ex Beal) Valdés-Reyna & Barkworth, Contr. U.S. Natl. Herb. 48: 17. 2003. Type: Mexico, Chihuahua, Santa Eulalia Mountains, 14 Aug 1885, *C.G. Pringle 385* (holotype: MSC-0092939 [image!]; isotypes: AC-00320221 [image!], BM-000938477 [image!], BR-0000006884895 [image!], JE-00001162 [image!], G-00168339 [image!], G-00168541 [image!], G-00168542 [image!], G-00168543 [image!], GH-00024478 [image!], K-000433421 [image!], KFTA-0000585 [image!], MO-123114 [image!], MO-123115 [image!], MO-5114652, NY-00431585 [image!], NY-00431586 [image!], NY-00431587 [image!], US-90985!, US-155154!, US-825176!, W-19160026109 [image!]).

### Ptilagrostiella

Taxon classificationPlantaePoalesPoaceae

Romasch., P.M.Peterson & Soreng, gen. nov.

urn:lsid:ipni.org:names:77199104-1

#### Type.

*Ptilagrostiellakingii* (Bol.) Romasch. (≡ *Stipakingii* Bol.).

#### Diagnosis.

*Ptilagrostiella* differs from *Piptatheropsis* Romasch., P.M. Peterson & Soreng in having glumes without veins with obtuse apices, a sharp callus, and laterally compressed florets with lemma margins overlapping most of the palea at maturity; and differs from *Ptilagrostis* Griseb. in having a sharp-pointed callus and lemmatal awns with very short hairs.

#### Description.

Plants perennial, cespitose, not rhizomatous with intravaginal branching. Culms 15–40 cm tall, 0.4–0.8 mm in diameter, erect, glabrous, not branching above. Leaf sheaths open, glabrous to scaberulous; ligules 1–2.5 mm long, membranous, apex obtuse to acute; blades 3–15 cm long, 0.3–0.5 mm wide, convolute, filiform, flexuous. Panicles 4–10 cm long, loosely contracted; branches ascending and usually appressed. Spikelets 3–4.5 mm long, lanceolate with one fertile floret without rachilla extension; disarticulation above the glumes; glumes 3–4.5 mm long, usually longer than the florets, hyaline, without veins, apex obtuse; florets 2.8–4.2 mm long, laterally compressed; calluses 0.3–0.7 mm long, sharp, hairy; lemmas 2.8–4.2 mm long, membranous to chartaceous, evenly pubescent throughout, the hairs 0.3–0.5 mm long, margins overlapping most of the palea at maturity, apex 2-lobed, the lobes 0.1–0.4 mm long, awned; lemma epidermal pattern saw-like; fundamental cells of variable length with sinuous sidewalls 2–8 times longer than silica cells irregularly alternating; silica bodies elongated-rectangular, sometimes paired with square-shaped cork cells; lemmatal awns 10–14 mm long, strigillose in lower part; 1- or 2-geniculate, persistent; paleas 2.6–3.2 mm long, shorter to about as long as the lemma, chartaceous, 2-veined; stamens 3, anthers 0.5–1.5 mm long, penicillate; lodicules 3, membranous; stigmas 2; ovary glabrous. Caryopses 1.5–2.3 mm long, fusiform, pericarp adherent. Chromosome number 2*n* = 22 (Johnson 1945).

### Ptilagrostiella kingii

Taxon classificationPlantaePoalesPoaceae

(Bol.) Romasch., comb. nov.

urn:lsid:ipni.org:names:77199105-1

Stipa kingii Bol., Proc. Calif. Acad. Sci. 4: 170. 1872 [Basionym] ≡ Oryzopsiskingii (Bol.) Beal, Grass. N. Amer. 2: 229. 1896 ≡ Ptilagrostiskingii (Bol.) Barkworth, Syst. Bot. 8(4): 417. 1983. Type: USA, California, Tuolumne Co., Mt. Dana and Toulumne Meadows, 7000–12000 ft, Sep 1866, *H.N. Bolander 6097* (lectotype: CAS-0005666 [image!] designated by M.E. Barkworth, Syst. Bot. 8: 417. 1983; isolectotypes: BM-001042147 [image!], F-0047023F [image!], G-00176575 [image!], G-00176576 [image!], G-00176577 [image!], GH-00361770 [image!, NY-01785914 [image!], US-81910!, YU-000920 [image!], YU-244788 [image!], W-18890217500 [image!]). Fig. 4F–J.

#### Distribution and habitat.

*Ptilagrostiellakingii* is endemic to California known only in the Sierra Nevada (Fresno, Inyo, Madera, Mariposa, Mono, Tulare, and Tuolomne counties) and is associated with lodgepole and subalpine forests (Calflora 2018). The species grows along moist streambanks and open, wet to dry meadows; 2000–3650 m (Barkworth 1983, 2007).

#### Comments.

In our earlier molecular analysis, *Ptilagrostiellakingii* is sister to a well-supported clade of *Piptatheropsis* (Romaschenko et al. 2011, 2012). As indicated by Barkworth (1983), the similarities between *P.kingii* and *Ptilagrostis* may have resulted from convergent evolution in distantly related taxa growing under similar environmental conditions since the former species shares an immediate common ancestor with *Piptatheropsis* and does not align near the *Ptilagrostis* clade (Romaschenko et al. 2012). *Ptilagrostiellakingii* also lacks a blunt callus and the plumose awns characteristic of most *Ptilagrostis* species (Wu and Phillips 2006).

### Thorneochloa

Taxon classificationPlantaePoalesPoaceae

Romasch., P.M.Peterson & Soreng, gen. nov.

urn:lsid:ipni.org:names:77199107-1

#### Type.

*Thorneochloadiegoensis* (Swallen) Romasch. ≡ (*Stipadiegoensis* Swallen).

#### Diagnosis.

*Thorneochloa* differs from *Pseudoeriocoma* Romasch., P.M. Peterson & Soreng in having dense pubescence 3–9 mm below the lower nodes, the hairs retrorse, non ramified branching on the middle and upper culms, and pedicels usually shorter than the spikelets.

#### Description.

Plants perennial, cespitose, not rhizomatous. Culms 70–140 cm tall, erect or ascending, often geniculate, 2–4 mm thick never bamboo-like or ramified above with (2) 3 nodes that are densely pubescent 3–9 mm below the lower nodes, the hairs retrorse, internodes usually pubescent. Leaf sheaths longer than the internodes below and shorter than the internodes above, glabrous or pubescent, ciliate on the margins and summit; collars with a tuft of hairs, the hairs 1.5–2 mm long; ligules 1–3 mm long, membranous and pubescent, apex truncate to obtuse; blades 15–40 cm long, 1–3.5 mm wide, flat to involute, scabrous below and pubescent above. Panicles 15–30 cm long, (2–) 4–8 cm wide, narrow, densely flowered, branches ascending appressed; pedicles usually shorter than the spikelets. Spikelets 8–11.5 mm long, lanceolate with one fertile floret without rachilla extension; disarticulation above the glumes; glumes 8–11.5 mm long, longer than the florets, subequal, hyaline, 3–5-veined, glabrous, acuminate; florets 5.5–7.5 mm long, usually fusiform; calluses 0.25–1.2 mm long, sharp, hairy; lemmas 5.5–7.5 mm long, fusiform, coriaceous, evenly hairy, the hairs 0.5–2 mm long, margins enveloping most of the palea, apex awned with minute apical lobes 0.2–0.4 mm long; lemma epidermal pattern maize-like; fundamental cells squared, longitudinally compressed with straight thin sidewalls subequal to silica cells (silica bodies) or shorter, regularly alternating; cork cells absent; lemmatal awns 20–50 mm long, 2-geniculate, flexuous, the segments scabrous, terminal segment straight; paleas 2.6–4 mm long, 1/2 to ¾ as long as the lemmas, 2-veined, veins not prolonged, hairy; anthers 2.5–4 mm long, not penicillate, 3 in number; lodicules 2 or 3; stigmas 2. Caryopses 3.8–4 mm long, fusiform, pericarp adherent, hilum linear, embryo ¼ the length.

#### Etymology.

The generic name honors Robert Folgers Thorne (1920–2015), an American taxonomist who specialized in the evolution and classification of vascular plants, known as the Thorne system.

### Thorneochloa diegoensis

Taxon classificationPlantaePoalesPoaceae

(Swallen) Romasch., comb. nov.

urn:lsid:ipni.org:names:77199108-1 77199108-1

Stipa diegoensis Swallen, J. Wash. Acad. Sci. 30(5): 212, f. 2. 1940 [Basionym] ≡ Achnatherumdiegoense (Swallen) Barkworth, Phytologia 74(1): 7. 1993. Type: USA, California, San Diego Co., Proctor Valley near Jamul, along vernal stream in chaparral, 23 May 1938, *F. F. Gander 5778* (holotype: US-1761177!; isotypes: AHUC-30095 [image!], CAS-0005662 [image!], DAO-000465418 [image!], F-0044439F [image!], SD-00000072 [image!]). Fig. 5A–E.

#### Distribution and habitat.

*Thorneochloadiegoensis* is found in Channel Islands (Santa Barbara County), San Diego, and Ventura Counties and Baja California, Mexico in rocky soil along vernal streams and canyons in chaparral and coastal sage-scrub vegetation; usually below 500 m (Barkworth 2007; Calflora 2018).

#### Comments.

Molecular sequence analysis reveals multiple origins of this taxon. In our preliminary ITS-derived phylogenetic tree *Thorneochloadiegoensis* aligns within *Nassella* whereas in the combined plastid-derived tree it aligns within *Pseudoeriocoma* (Valdés Reyna et al. 2013). Geographically, the most likely parents, if of hybrid origin, would be *Nassellamucronata* (Kunth) R.W. Pohl and *Pseudoeriocomaeminens*. A more detailed genetic study using low-copy nuclear genes would perhaps resolve this hypothesis.

##### A key to the native and introduced (marked with an asterisk) genera of Stipeae (and Ampelodesmeae) in North America (modified from Barkworth 2007)

**Table:** 

1	Spikelets with 2–6 florets; cultivated as ornamental, a Mediterranean species escaped in California	*Ampelodesmos* Link^*^ (tribe Ampelodesmeae)
–	Spikelets with 1 floret (tribe Stipeae); plants native or not	**2**
2	Paleas sulcate, longer than the lemmas; lemma margins involute, fitting into the paleal grove; lemma apices not lobed	***Piptochaetium***
–	Paleas flat, from shorter than to longer than the lemmas; lemma margins convolute or not overlapping; lemma apices often lobed or bifid	**3**
3	Prophylls exceeding the leaf sheaths; lemmas with 2 prominent lobes at apex (0.9–2 mm long); plants cultivated as ornamentals, not escaped	**4**
–	Prophylls concealed by the leaf sheaths; lemmas with mostly shorter lobed or unlobed apices; plants native, introduced from Mediterranean region, sometimes cultivated as ornamentals	**5**
4	Panicles contracted; lemma awns once-geniculate, first segment plumose; style 1	***Macrochloa*** ^*^
–	Panicles open; lemma awns twice-geniculate, segments glabrous; styles 2	***Celtica*** ^*^
5	Plants with multiple stiff branches from the upper nodes; pedicels sometimes plumose; Australian species cultivated as ornamentals in the Flora region	Austrostipa ^*^
–	Plants not branching at the upper nodes, or with a few, flexible branches (*Pseudoeriocoma*); pedicels never plumose; species native, established introductions, or cultivated as ornamentals	**6**
6	Apices of the leaf blade sharp and stiff; caryopses obovoid, often with 3 smooth ribs at maturity; cleistogenes usually present in sheaths; plants adventive in California, native from Mexico southward	***Amelichloa***
–	Apices of the leaf blades acute to acuminate, never both sharp and stiff; caryopses fusiform, ovoid or obovoid, without ribs; cleistogenes sometimes present in sheaths	**7**
7	Lemma margins strongly overlapping over their whole length at maturity, lemma bodies usually rough throughout, apices with a membranous or indurate crown and not lobed; paleas ¼–½ the length of the lemmas, without veins, glabrous; plants native to North America and southward, South American species sometimes cultivated as ornamentals and escaped	***Nassella***
–	Lemma margins usually not or only slightly overlapping for some or all of their length at maturity, strongly overlapping in some species with smooth lemmas, lemma bodies usually smooth on the lower portion, apices often 1–2-lobed and never with a membranous or indurate crown; paleas from 1/3 as long as to equaling or slightly exceeding the lemmas, 2-veined at least on the lower portion, usually with hairs or both lemmas and paleas glabrous	**8**
8	Calluses 1.5–6 mm long, sharply pointed; plants perennial or annual, if perennial, awns 65–500 mm long, if annual, awns 50–100 mm long; panicle branches straight	**9**
–	Calluses 0.1–2 mm long, blunt to sharply pointed; plants perennial; awns 1–70 mm; panicle branches straight or flexuous	**12**
9	Lower ligules densely hairy, upper ligules less densely hairy or glabrous; awns plumose in lower segment, glabrous above, unigeniculate; plants perennial	***Pappostipa***
–	Ligules glabrous or inconspicuously pubescent, lower and upper ligules alike in vestiture; awns glabrous or pilose throughout or in lower segment; plants perennial or annual	**10**
10	Plants perennial; florets 7–25 mm long; awns scabrous or pilose on the first 2 segments, the terminal segment scabrous, or if pilose, the hairs 1–3 mm long	***Hesperostipa***
–	Plants annual or perennial, if perennial, the florets 18–27 mm long and the awns plumose on the terminal segment, the hairs 5–6 mm long	**11**
11	Plants annual; glumes 12–20 mm long; florets 4–7 mm long; awn sparsely short hairy in basal segment only; plants adventive from Mediterranean, noxious weeds in Southern California	Stipelulla^*^
–	Plants perennial (sometimes short-lived); glumes 60–90 cm long; florets 18–27 mm long; the awns plumose on the terminal segment, the hairs 5–6 mm long; plants cultivated ornamentals from Eurasia, not escaped	Stipa ^*^
12	Panicles to 60 cm long, delicate, nodding, branches capillary, loosely spreading to spreading in distant whorls; lemmas 2 mm long, coarsely scabrous distally, margins meeting or slightly gapped; callus with a brief ring of hairs; awns caducous, to 8 mm long, slender, scabrous, curved; anther 1, 0.8–1.4 mm long, apically thickened, not penicilliate; plants cultivated ornamentals from New Zealand, not escaped	Anemanthele ^*^
–	Panicles of various lengths, and shapes (similar in *Oloptum*, but lemma surfaces smooth, margins widely gapped in middle and fused at base, callus glabrous); lemmas usually longer; awns various; anthers 3, not apically thickened, penicillate or not; plants sometimes cultivated	**13**
13	Florets usually dorsally compressed at maturity, sometimes terete; paleas as long as or longer than the lemmas and similar in texture and pubescence; lemma margins separate for their whole length at maturity	**14**
–	Florets terete or laterally compressed at maturity; paleas often shorter than the lemmas, sometimes less pubescent, sometimes as long as the lemmas and similar in texture and pubescence; lemma margins often overlapping for part or all of their length at maturity	**17**
14	Callus barbed with a dense ring of flexuous hairs, hairs 1.0–1.5 mm long; style 1; lodicules 2; elongated leaf blades concentrated basally (above initial cataphylls), upper cauline leaves much reduced, only 0.8–1.8 cm long; lemma epidermal pattern saw-like	***Oryzopsis***
–	Callus glabrous or with short straight hairs forming a sparse ring, hairs 0.1–0.5 mm long; styles 2; lodicules 2 or 3; awn central; cauline leaves well developed, similar to basal leaves, or somewhat shorter but not strongly reduced; lemma epidermal pattern saw-like or maize-like	**15**
15	Glumes 5–9-veined, with faint or prominent transverse veinlets; basal leaf blades absent (leaves cataphyllous) then up to 2 cm long; mid- and upper cauline leaves several, up to 35 cm long and 2 cm wide	***Patis***
–	Glumes 1–3-veined, transverse veinlets absent (rarely present, never prominent); basal leaf blades well developed or not (leaves cataphyllous or not), mostly 2–90 cm long or reduced; cauline leaves similar to basal leaves, or sometimes shorter or rudimentary	**16**
16	Plants with well-developed basal tufts leaves, blades slender; central vein of the lemma not prominent; lower panicle branches never whorled; anther apices glabrous; lemma epidermal pattern Saw-like; awns caducous and straight and basally slightly twisted, or persistent and geniculate with a strongly twisted first segment; plants native	***Piptatheropsis***
–	Plants without basal tufts of leaves, blades 2–10 mm wide; central vein of the lemma prominent; lower panicle branches whorled with 3‒30 or more per node; anther apices minutely bearded; lemma epidermal pattern Maize-like; awns persistent or caducous, straight, never twisted; plants adventive from Eurasia	Oloptum ^*^
17	Glumes without evident venation, glume apices rounded to acute; plants subalpine to alpine, sometimes growing in bogs	**18**
–	Glumes with 1–3(5) evident veins or the glume apices attenuate; plants growing from near sea level to subalpine or alpine habitats, not growing in bogs	**19**
18	Awns strigillose in lower part; lemma lobes inconspicuous (0.1–0.4 mm); callus sharp; panicles narrow to loosely contracted; anthers penicillate, 0.5–1.5 mm long	***Ptilagrostiella***
–	Awns hairy throughout, lemma lobes prominent (up to 0.8 mm); callus blunt; the hairs on the lowest segment 1–2 mm long; panicles open with spreading branches these sometimes loosely contracted; anthers glabrous, 1.2–3 mm long	***Ptilagrostis***
19	Paleas with prolonged veins almost reaching the tip of the lemma lobes, the veins 1–3 mm long; lemma apices 2-lobed, narrow, the lobes 1–3 mm long	***Barkworthia***
–	Paleas without prolonged veins or if prolonged never more than 0.3 mm long; lemma apices unlobed or if lobed, the lobes usually obtuse and never more than 2.1 mm long	**20**
20	Lemma bodies with hairs to 0.15 mm long over most of their length, and a tuft of pappus-like hairs at the apex to 3–4 mm long; awns glabrous; ligules with lateral tufts of hairs to 2 mm long; anthers 0.8 mm long; plants native from Mexico southward, infrequently cultivated as an ornamental	***Jarava***
–	Lemma bodies with evenly distributed hairs of similar length or completely glabrous, sometimes with longer hairs around the base of the awn; basal segment of the awns sometimes with hairs up to 2 mm long; ligules without lateral tufts of hairs; anthers mostly longer; plants of Mexico and northward, infrequently cultivated as an ornamentals	**21**
21	Basal leaf sheaths becoming fibrous with age; panicle branches whorled below; apical lemma hairs 1–1.5 mm long; awns readily deciduous; upper culm ligules to 12 mm long; plants cultivated ornamentals from Asia, uncommon, not known to have escaped	Neotrinia ^*^
–	Basal leaf sheaths never fibrous, occasionally ribbon-like; panicle branches rarely whorled below; lemmas usually without apical lemmas hairs longer than those present on the body; upper culm ligules usually less than 5 mm long; plants native and widespread	**22**
22	Plants with woody, sometimes scandent bamboo-like culms, 3–6 mm thick below with ramified branching (usually, but sometimes absent in immature specimens of *P.hirticulmis*) at the middle and upper nodes, with (2) 3 to 13 nodes	***Pseudoeriocoma***
–	Plants with neither woody nor scandent bamboo-like culms, usually less than 2 mm thick below and never with ramified branching at the middle and upper nodes, with 2 to 3 or up to 5 nodes in a few species	**23**
23	Lower culm internodes densely pubescent for 3–9 mm below the nodes, the hairs retrorse with shorter hairs and less densely pubescent elsewhere; known only from southern California and Baja California	***Thorneochloa***
–	Lower culm internodes glabrous or if pubescent then only to 5 mm below the nodes, usually glabrous elsewhere or if hairy the hairs usually not retrorse; widely distributed in western North America	***Eriocoma***

##### Excluded name

### Stipa virlettii

Taxon classificationPlantaePoalesPoaceae

E. Fourn., Mexic. Pl. 2: 75. 1886.

#### Comments.

The description of *Stipavirlettii* (Type: *Virlet 1376* from San Luis de Potosí, Mexico) appears to be a mixture of *Stipamucronata* Kunth [= *Nassellamucronata*] awns and *Bromuslaciniatus* Beal (= *Bromuscarinatus* Hook & Arn.) as determined by A.S. Hitchcock (isotype: US-A866077 fragm. ex P-Fourn-163!). Notes by A.S. Hitchcock on the US sheet with two fragment packets indicate that there are two species of *Bromus* on the herb. Fournier sheet: A is *B.richardsonii* Link; B is *B.carinatus*, also annotated by ASH.

## Supplementary Material

XML Treatment for Barkworthia

XML Treatment for Barkworthia stillmanii

XML Treatment for Eriocoma

XML Treatment for Eriocoma alta

XML Treatment for Eriocoma arida

XML Treatment for Eriocoma arnowiae

XML Treatment for Eriocoma bloomeri

XML Treatment for Eriocoma bracteata

XML Treatment for Eriocoma contracta

XML Treatment for Eriocoma coronata

XML Treatment for Eriocoma curvifolia

XML Treatment for Eriocoma hendersonii

XML Treatment for Eriocoma hymenoides

XML Treatment for Eriocoma latiglumis

XML Treatment for Eriocoma lemmonii

XML Treatment for Eriocoma lemmonii subsp. pubescens

XML Treatment for Eriocoma lettermanii

XML Treatment for Eriocoma lobata

XML Treatment for Eriocoma nelsonii

XML Treatment for Eriocoma nelsonii subsp. dorei

XML Treatment for Eriocoma nevadensis

XML Treatment for Eriocoma occidentalis

XML Treatment for Eriocoma occidentalis subsp. californica

XML Treatment for Eriocoma occidentalis subsp. pubescens

XML Treatment for Eriocoma parishii

XML Treatment for Eriocoma parishii subsp. depauperata

XML Treatment for Eriocoma perplexa

XML Treatment for Eriocoma pinetorum

XML Treatment for Eriocoma richardsonii

XML Treatment for Eriocoma robusta

XML Treatment for Eriocoma scribneri

XML Treatment for Eriocoma swallenii

XML Treatment for Eriocoma thurberiana

XML Treatment for Eriocoma wallowaensis

XML Treatment for Eriocoma webberi

XML Treatment for Eriosella

XML Treatment for Eriosella caduca

XML Treatment for Neotrinia

XML Treatment for Neotrinia splendens

XML Treatment for Oloptum

XML Treatment for Oloptum miliaceum

XML Treatment for Pseudoeriocoma

XML Treatment for Pseudoeriocoma acuta

XML Treatment for Pseudoeriocoma constricta

XML Treatment for Pseudoeriocoma editorum

XML Treatment for Pseudoeriocoma eminens

XML Treatment for Pseudoeriocoma hirticulmis

XML Treatment for Pseudoeriocoma multinodis

XML Treatment for Ptilagrostiella

XML Treatment for Ptilagrostiella kingii

XML Treatment for Thorneochloa

XML Treatment for Thorneochloa diegoensis

XML Treatment for Stipa virlettii

## References

[B1] Barkworth ME Maze J (1979) Proposal to reject *Stipacolumbiana* (Poaceae) and nomenclatural changes affecting three western North American species of *Stipa* (Poaceae). Taxon 28(5/6): 621–624. 10.2307/1219826

[B2] Barkworth ME (1983) *Ptilagrostis* in North America and its relationship to other Stipeae. Systematic Botany 8(4): 395–419. 10.2307/2418359

[B3] Barkworth ME (2007) 10. Stipeae Dumort. In: Barkworth ME Capels KM Long S Anderton LK Piep MB (Eds) Magnoliophyta: Commelinidae (in part): Poaceae, part 1.Oxford Universtiy Press, New York, 109–186.

[B4] Barkworth ME Everett J (1988) Evolution in Stipeae: Identification and relationships of its monophyletic taxa. In: Soderstrom TR Hilu KW Campbell CS Barkworth ME (Eds) Grass Systematics and Evolution.Smithsonian Institution Press, Washington, D.C., 251–264.

[B5] Calflora (2018) Information on California plants for education, research and conservation. Berkeley, California: The Calflora Database (a non-profit organization). https://www.calflora.org/ [accessed 27 Dec 2018]

[B6] Dávila P Mejia-Saulés MT Soriano-Martínez AM Herrera-Arrieta Y (2018) Conocimiento taxonómico de la familia Poaceae en México. Botanical Sciences 96(3): 462–514. 10.17129/botsci.1894

[B7] Devesa JA Ruiz T Viera MC Tormo R Vázquez F Carrasco JP Ortega A Pastor J (1991) Contribución al conocimiento cariológico de las Poaceae en extremadura (España)–III. Boletim da Sociedade Broteriana, sér. 2 64: 35–74.

[B8] Espejo Serna A López-Ferrari AR Valdés-Reyna J (2000) Poaceae. In: Espejo Serna A López-Ferrari AR (Eds) Las Monocotyledóneas Mexicanus: una synopsis florística, Partes IX–XI.Consejo Nacional de la Flora de México, A.C., Univerisidad Autónoma Metropolitana-Izapalapa, and Comisión Nacional para el conocimiento & uso de la Biodiversidad, México, 10, 8–236.

[B9] Faruqi SA Quraish HB Inamuddin M (1987) Studies in Libyan grasses X. Chromosome number and some interesting features. Annals of Botany (Rome) 45: 75–102.

[B10] Freitag H (1975) The genus *Piptatherum* (Gramineae) in Southwest Asia. Notes from the Royal Botanic Garden Edinburgh 33: 341–406.

[B11] Freitag H (1985) The genus *Stipa* (Gramineae) in Southwest and South Asia. Notes from the Royal Botanic Garden Edinburgh 42: 355–489.

[B12] Gleason HA Cronquist A (1991) Manual of vascular plants of northeastern United States and adjacent Canada. The New York Botanical Garden, Bronx, New York, 910 pp.

[B13] Gohil RN Koul KK (1986) SOCGI plant chromosome number reports – IV. Journal of Cytological Genetics 21: 155.

[B14] Hamasha HR Bernhard von Hagen K Röser M (2012) *Stipa* (Poaceae) and allies in the Old World: Molecular phylogenetics realigns genus circumscription and gives evidence on the origin of American and Australian lineages. Plant Systematics and Evolution 298(2): 351–367. 10.1007/s00606-011-0549-5

[B15] Hitchcock AS (1935) 96. *Stipa*, 97. *Oryzopsis*, and 98. *Piptochaetium*. North American Flora 17: 406–431.

[B16] Hitchcock AS (1951) Manual of Grasses of the United States (revised by A. Chase). US Department of Agriculture Miscellaneous Publication 200: 1–1051. 10.5962/bhl.title.65332

[B17] Ibrahim KM Hosni HA Peterson PM (2016) Grasses of Egypt. Smithsonian Contributions to Botany 103: 1–201. 10.5479/si.19382812.103

[B18] Johnson BL (1945) Natural hybrids between *Oryzopsishymenoides* and several species of *Stipa*. American Journal of Botany 32(9): 599–608. 10.1002/j.1537-2197.1945.tb05164.x

[B19] Johnson BL Rogler GA (1943) A cyto-taxonomic study of an intergeneric hybrid between *Oryzopsishymenoides* and *Stipaviridula*. American Journal of Botany 30(1): 49–56. 10.1002/j.1537-2197.1943.tb14731.x

[B20] Luque T Lifante ZD (1991) Chromosome numbers of plants collected during Iter Meditrraneum I in the SE of Spain. Bocconea 1: 303–364.

[B21] Nobis M Gudkova PD Nowak A (2019) *Neotrinia* gen. nov. and Pennatherum sect. nov. in Achnatherum (Poaceae: Stipeae). Turczaninowia 22(1): 37–41. 10.14258/turczaninowia.22.1.5

[B22] Romaschenko K Garcia-Jacas N Peterson PM Soreng RJ Vilatersana R Susanna A (2013) Miocene–Pliocene speciation, introgression, and migration of *Patis* and *Ptilagrostis* (Poaceae: Stipeae). Molecular Phylogenetics and Evolution 70(2014): 244–259. 10.1016/j.ympev.2013.09.018 24096057

[B23] Romaschenko K Peterson PM Soreng RJ Garcia-Jacas N Futoma O Susanna A (2008) Molecular phylogenetic analysis of the American Stipeae (Poaceae) resolves *Jarava* sensu lato polyphyletic: Evidence for a new genus, *Pappostipa*. Journal of the Botanical Research Institute of Texas 2: 165–192.

[B24] Romaschenko K Peterson PM Soreng RJ Garcia-Jacas N Susanna A (2010) Phylogenetics of Stipeae (Poaceae: Pooideae) based on plastid and nuclear DNA sequences. In: Seberg O Petersen G Barfod AS Davis JI (Eds) Diversity, phylogeny, and evolution in the monocotyledons.Aarhus University Press, Denmark, 513–539.

[B25] Romaschenko K Peterson PM Soreng RJ Futorna O Susanna A (2011) Phylogenetics of *Piptatherum* s.l. (Poaceae: Stipeae): Evidence for a new genus, *Piptatheropsis*, and resurrection of *Patis*. Taxon 60(6): 1703–1716. 10.1002/tax.606015

[B26] Romaschenko K Peterson PM Soreng RJ Garcia-Jacas N Futorna O Susanna A (2012) Systematics and evolution of the needle grasses (Poaceae: Pooideae: Stipeae) based on analysis of multiple chloroplast loci, ITS, and lemma micromorphology. Taxon 61(1): 18–44. 10.1002/tax.611002

[B27] Romaschenko K Peterson PM Soreng RJ Valdés Reyna J (2014) A molecular phylogeny and classification of *Eriocoma* and *Ptilostipa* (Poaceae: Stipeae). Botany2014, New Frontiers in Botany, Systematics Section, held at the Boise Convention Centre, Boise, Idaho (Abstract 26–31 July 2014) http://2014.botanyconference.org/engine/search/index.php?func=detail&aid=233

[B28] Roshevitz RJ (1951) De genere *Piptatherum* P.B. notae criticae. Botanicheskie materialy Gerbariya Botanicheskogo Instituti imeni V. L. Komarova, Akademii Nauk SSSR (Leningrad) 14: 78‒129.

[B29] Sánchez-Ken JG (2018) Riqueza de especies, clasificación y listado de las gramíneas (Poaceae) de México. Acta Botánica Mexicana 126: 1–115. 10.21829/abm126.2019.1379

[B30] Soreng RJ Peterson PM Davidse G Judziewicz EJ Zuloaga FO Filgueiras TS Morrone O (2003) Catalogue of New World grasses (Poaceae): IV. subfamily Pooideae. Contributions from the U.S. National Herbarium 48: 1–730. http://botany.si.edu/pubs/CUSNH/ContList.htm

[B31] Soreng RJ Peterson PM Romaschenko K Davidse G Zuloaga FO Judziewicz EJ Filgueiras TS Davis JI Morrone O (2015) A worldwide phylogenetic classiﬁcation of the Poaceae (Gramineae). Journal of Systematics and Evolution 53(2): 117–137. 10.1111/jse.12150

[B32] Soreng RJ Peterson PM Romaschenko K Davidse G Teisher JK Clark LG Barberá P Gillespie LJ Zuloaga FO (2017) A worldwide phylogenetic classification of the Poaceae (Gramineae) II: An update and a comparison of two 2015 classifications. Journal of Systematics and Evolution 55(4): 259–290. 10.1111/jse.12262

[B33] Thomasson JR (1978) Epidermal patterns of the lemma in some fossil and living grasses and their phylogenetic significance. Science 199(4332): 975–977. 10.1126/science.199.4332.975 17752369

[B34] Thomasson JR (1980) *Paleoeriocoma* (Gramineae, Stipeae) from the Miocene of Nebraska: Taxonomic and phylogenetic significance. Systematic Botany 5(3): 233–240. 10.2307/2418370

[B35] Thomasson JR (1981) Micromorphology of the lemma in *Stiparobusta* and *Stipaviridula* (Gramineae: Stipeae): Taxonomic significance. The Southwestern Naturalist 26(2): 211–214. 10.2307/3671126

[B36] Thomasson JR (1982) Fossil grass anthoecia and other plant fossils from arthropod burrows in the Miocene of Western Nebraska. Journal of Paleontology 56: 1011–1017.

[B37] Thomasson JR (1985) Miocene fossil grasses: Possible adaptation in reproductive bracts (lemma and palea). Annals of the Missouri Botanical Garden 72(4): 843–851. 10.2307/2399226

[B38] Tzvelev NN (1974) Notes of the tribe Stipeae Dum., family Poaceae in URSS. Novosti Sistematiki Vysshchikh Rastenii 11: 4–21.

[B39] Tzvelev NN (1976) Zlaki SSSR [Grasses of the Soviet Union]. Nauka Publishers USSR, Leningrad.

[B40] Tzvelev NN (1977) On the origin and evolution of feathergrasses (*Stipa* L.). In: Karamysheva ZV (Ed.) Problemy ekologii, geobotaniki, botanicheskoi geografii i floristiki.Academiya Nauk SSSR, Leningrad, 139–150.

[B41] Tzvelev NN (2012) Notes on the tribe Stipeae Dumort. (Poaceae). Novosti Sistematiki Vysshchikh Rastenii 43: 20–29.

[B42] Valdés Reyna J (2015) Gramíneas de Coahuila. Comisión Nacional para el Conocimiento y Uso de la Biodiversidad, Mexico, 557 pp.

[B43] Valdés Reyna J Romaschenko K Peterson PM Soreng RJ (2013) A molecular phylogeny and classification of *Eriocoma* and *Pseudoeriocoma* (Poaceae: Stipeae). Monocots V, 5^th^ International Conference on Comparative Biology of Monocotyledons, New York, held at Fordham University Campus, Keating Hall Auditorium (Abstract 8 July 2013). https://www.regonline.com/builder/site/Default.aspx?EventID=1060172

[B44] Verlaque R Reynaud C Aboucaya A (1997) Mediterranean chromosome number reports 7 (843–854). Flora Mediterranea 7: 240–246.

[B45] Watson L Dallwitz M (1992) The Grass Genera of the World. CAB International, Wallingford, 1038 pp.

[B46] Wu ZL Phillips SM (2006) 7. Tribe Stipeae. In: Wu Z Raven PH Hong DY (Eds) Flora of China, Poaceae.Science Press and Missouri Botanical Garden Press, Beijing and St. Louis, 188–212.

